# Uncovering hidden patterns: How good governance and health outcomes are shaped by the public healthcare funding?

**DOI:** 10.3389/fpubh.2026.1925604

**Published:** 2026-07-30

**Authors:** Alexandra-Mădălina Țăran, Mihaela Diaconu, Anamaria Vasiu, Marilen-Gabriel Pirtea

**Affiliations:** 1 Department of Finance, Information System and Business Modelling, Faculty of Economics and Business Administration, West University of Timisoara, Timisoara, Romania; 2 Faculty of Economics and Business Administration, West University of Timisoara, Timisoara, Romania

**Keywords:** chord diagrams, cross-country analysis, Gaussian Graphical Models (GGMs), health governance, healthcare financing, Ward’s method

## Abstract

**Introduction:**

This study aims to empirically investigate the relationships between governance quality and health outcomes, considering the mediating role of healthcare funding.

**Methods:**

The Gaussian Graphical Models (GGM) and the Ward method (hierarchical clustering) were used to investigate the relationship between good governance and health status, considering health expenditure as a key pathway to bridge health outcomes. This study comprises panel data covering the period from 2008 to 2024.

**Results:**

Good quality of public governance demonstrates a stronger positive impact on the health status at higher level of health financing, with this effect diminishing progressively across the distribution to lower allocation of health expenditures by the governments. Health financing exhibits a consistently robust positive relationship with the health status throughout the obtained interlinkages, remaining statistically significant across all inferences examined. The results display differences among the cross-country analysis that highlight the need to strengthen the governance administrative framework and ensure efficient management of financial resources to support the performance of healthcare systems. Furthermore, the results suggest the importance of implementing public strategies tailored to the emerging needs of the population and to focus on the developing health systems by supporting prevention, the digitalization of medical services, reduce health-related costs, and improving the population’s access to quality medical services.

**Discussion:**

Moreover, streamlining the mechanisms for the allocation of financial resources can help reduce disparities between countries and improve the performance of healthcare systems. The findings contribute to the existing specialized body of knowledge on sustainable health systems and provide practical guidance for policymakers seeking to promote adapted health policies as regards the transition risk while safeguarding good health of the citizens.

## 1 Introduction

Nowadays the health system represents a key feature of social stability, particularly due to the fact that is considered to play a vital role in ensuring the well-being of the population and in managing different crisis situations. Nevertheless, the unprecedented shocks during the COVID-19 pandemic have highlighted the need for effective public governance mechanisms, adequate strategies for funding, and the institutional capacity to adapt promptly to external pressures on the healthcare sector.

Within the context of the rapid increase in the number of cases and the growing pressure on medical infrastructure, the European Member States were compelled to implement imperative measures aimed at reconfiguring medical services and leveraging the existing resources. In the context of insufficient resources, effective coordination of decisions and ensuring the population’s access to quality services contributed significantly to maintaining the stability of health systems and reducing the impact on individuals (1). Nevertheless, public governance reflects a major factor influencing the quality of services and the performance of public institutions. In the context of urbanization and economic development, the efficiency of public administration is a key factor in ensuring access to quality public services and in improving the living conditions and health standards of the population (2–4). At the same time, in urban areas with a high level of economic development, citizens have better access to health care, education, and good hygiene conditions, factors that contribute to favorable health outcomes (5). Additionally, political instability, corruption, and a low governance performance can reduce the effectiveness of health policies and service delivery, leading to inadequate living conditions and dysfunction in the performance of the health systems (2, 6).

In the healthcare sector, its importance is amplified by the direct impact that administrative decisions exert on the well-being of the population. Thus, healthcare funding directly influences this relationship, affecting both the financial sustainability and the operation of healthcare services (7, 8). Furthermore, countries with a high level of economic development achieve favorable health outcomes, given to both financial resources and the quality of public governance. Countries characterized by low governance stability and high levels of corruption are often susceptible of dysfunction in the performance of their health systems.

Given that health systems are strongly affected by the effectiveness of institutional management and policy frameworks, rather than addressing the complete absence of research, this study extends the existing literature by examining the linkages between good public governance, healthcare outcomes, and health system financing within the European Union. Furthermore, the research aims to offer a hierarchy of EU-27 Member States, illustrated by the performance of public governance and how it further reflects on improving the level of funding directed toward the healthcare system, in order to highlight the diversity of public policies implemented, the existing regional disparities, as well as their effectiveness and performance across countries.

The research analysis employed advanced econometric techniques, involving a two-fold methodological approach designed to capture the complex and structural relationship between public sector governance, health outcomes, and public health funding. Accordingly, we seek to provide a complex review of the relevant literature that was conducted by applying the mapping technique, namely, bibliometric analysis performed using VOSviewer software, in order to identify past, present, and future emerging trends in health economics. The advanced empirical effort includes complex methods such as the Gaussian Graphical Model (GGM), applied in RStudio software, which consists of analyzing the conditional relationships between good governance, health status, and health financing, while the model estimation was performed based on partial correlations (PCOR) using the extended Bayesian information criterion (EBIC). Additionally, the final stage of the analytical process consists of applying hierarchical clustering, namely cluster analysis, developed in R software, aiming to identify the disparities among countries and to observe the registered level of high, medium or low performance in terms of public governance and healthcare system outcomes, implying also the moderating role of health financing. The empirical analysis was conducted using a panel dataset containing data for the European Union Member States for the period between 2008 and 2024, along with relevant indicators for the topic addressed, specifically the dimensions of governance, the healthcare sector, and health system financing.

Given the current context, defined by the many challenges the healthcare system has faced, especially during the COVID-19 pandemic, as well as geopolitical and global risks, improving the healthcare sector has emerged as a high priority for decision-makers in the European Union’s Member States. However, the existing specialized literature lacks a comprehensive research framework for examining the relationship between the quality of good governance and the mediating role of health system financing on citizens’ health status.

The present study brings a series of novel elements to the existing literature. One key original contribution is the holistic approach to public healthcare funding as a mediating mechanism between good governance and healthcare performance, expanding on the direct governance-healthcare relationship. Furthermore, the study offers a comprehensive perspective on the resilience of public health systems, as a result of integrating the COVID-19 pandemic and post-pandemic periods, thus analyzing the relationship between the three dimensions during periods of crisis, economic, and geopolitical external shocks. Furthermore, the empirical study implies a sophisticated methodological framework with modern econometric techniques, such as Chords diagrams, the Ward clustering analysis, and Gaussian Graphical Models based on the EBICglasso and PCOR information criteria, with the results supported by a series of robustness tests. The novelty of this research is also reflected by highlighting the heterogeneity of European economies through the classification of countries based on their performance across the analyzed dimensions, providing a useful framework for identifying best practices and public policy recommendations. The extended analysis of the specialized literature, which was conducted using two types of analysis: one bibliometric and the other systematic, led to the identification of the importance of governance for the performance of health systems and the identification of dominant research directions. At the same time, the exhaustive analysis of the literature revealed existing gaps in the literature, such as the fact that many authors provide only a partial analysis of the subject, including Hadipour et al. (9), who do not consider health system financing when analyzing the role of good governance in increasing life expectancy, while Onofrei et al. (10) do not consider the component of good governance in their study related to the health outcomes in the European healthcare sector. In addition to the review of multidisciplinary literature, the innovative nature of this paper is also reflected regarding the mixed-methods framework, which ensures the complexity of the study through a diversified empirical approach at both the panel and cross-sectional levels. Thus, a novel aspect that complements the study of Azimi et al. (11) is provided by the cross-sectional analysis using Ward’s hierarchical clustering, which allowed grouping the European countries and their comparison. Last but not least, our research contributes significantly to the existing literature, given that the timeframe covered includes not only the pandemic period but also the subsequent period characterized by numerous initiatives at the European level, such as EU4Health (2021–2027), the Recovery and Resilience Facility on which NextGenerationEU focuses, and the development of the European Health Union, measures taken to strengthen European health systems and prioritize health system funding and institutional resilience, under climate and geopolitical risks.

The Gaussian Graphical Models (GGMs) reveal a series of robust and significant interlinkages between the agents’ confidence in and abide by the rules of society, quality of public civil services and their degree of independence from political pressures, and the control of corruption, and their positive interlinkages with the resources that are allocated by governments in the health sector. Moreover, these results are further suggesting that administrative stability leads to a greater capacity to sustain medical infrastructure. In contrast, among the GGMs main results, we observe the negative and significant associations identified between infant mortality rate, life expectancy, and the inpatient beds available in hospitals, thus reflecting the existence of vulnerabilities in health status and the functioning of the medical system. Alongside, the main results of Ward’s method (hierarchical clustering) suggest that developed countries with high levels of governance quality can achieve favorable outcomes in the health sector, exhibiting positive inferences on the health status of the population, also emphasizing that the level of funding directly influences the performance of healthcare systems. In terms of countries with average or low performances as regards good governance and health, the findings reveal discrepancies in administrative quality, institutional structural deficiency, and inefficient resource management leads to a mitigation in the capacity of health systems to provide efficient and high-quality services. Ultimately, the implications drawn from the study emphasize the need to strengthen administrative structures to achieve favorable outcomes for public healthcare, as well as to redesign the allocation of financial resources to support the development of medical infrastructure and economic performance.

This study consists of several main sections: Section 1 introduces the research conceptual architecture, followed by Section 2 which design the theoretical framework through a systematic and bibliometric analysis of the specialized literature. The next section is Section 3, which applies two-fold advanced econometric methods to the analyzed dimensions. Section 4 and 5 highlights the main results and conclusions of our research.

## 2 Literature review and theoretical framework

In the last few decades, public governance has become a key factor in the quality of services and the performance of public institutions. It is particularly important in the health sector, where administrative decisions exhibit a direct impact on citizens’ well-being. Good governance is mainly considered as a complex process involving the exercise of authority, the coordination of relevant institutions, and the transparent and accountable management of resources. Financing different needs of healthcare generated the crucial link between the quality of governance and the outcomes of health systems, playing a very important role in the collection, allocation, and supervision and institutional control of funds, influencing both the financial sustainability and the performance of health services. In this context, many authors support the argument that the performance of health systems is particularly influenced by the quality of public institutions and their capacity to use public resources for the development of the healthcare sector (12–14). Recent research, through empirical studies, demonstrates the positive impact of the quality of governance on the population’s health as well as on the financing of healthcare systems. Ding et al. (15), based on their findings, confirm that countries that a high level of administrative efficiency has a positive effect on public health through innovation in the health and education sectors, as well as tackle corruption. Furthermore, good governance has a positive effect on supporting the development of medical infrastructure through the efficient allocation of public resources in the health sector (11), as further supported by the study by Onofrei et al. (10), which emphasized that a robust governance structure positively impacts the performance of health systems, and that increased public spending on health leads to significant improvements in life expectancy.

The role of financing in the performance of healthcare systems is a key factor both in reducing health risks and in ensuring equitable access to quality services in this sector (16), and the importance of this process emerges from its contribution to health sector, the improvement of the population’s well-being, and the support of economic development. Thus, financing systems based on taxes and mandatory contributions allow for expanded access to medical services and a reduction in the risks associated with exclusion from health systems. Since the 1970s, researchers have focused on the analysis of health expenditures, thus Newhouse (17) highlighted the influence of income on the level of medical expenditures.

As the good governance sets up the principles related to performance, transparency, accountability, and integrity, the public health represents one of the sectors where these principles translate into concrete outcomes. Thus, Osakede (18) confirms that the positive effect of healthcare funding on medical services and infrastructure can be ensured on a solid administrative structure effective in managing efficiently the existing resources, thereby contributing to the improvement of the population’s health. The study by Akintunde et al. (19) emphasizes that good governance contributes to improved health outcomes; it also recommends increasing health care spending in order to increase life expectancy. Similarly, Farag et al. (20) showed that the impact of government spending on health depends on the level of public governance, contributing significantly to the reduction of infant mortality as well as under-five mortality. This is further supported by the study by Hu and Mendoza (21), which argues that both institutional efficiency and the allocation of funds to healthcare support to reduce mortality among children under 1 year of age.

Many other researchers (22–28) reveal that the performance of healthcare systems is determined by the way in which funds are collected, distributed, and monitored along the complex processes. Nevertheless, performant financial governance is needed to be based on transparency, accountability, and public integrity, while weak performance health systems, lacks supervision and evaluation, leading to inefficiency, budget inefficiency, alongside social inequities (29). Gabani et al. (22) stated that the structure of health funding has a direct impact on health outcomes, revealing that states that have transitioned from models that are based on out-of-pocket payments toward government financing has observed an increase in life expectancy, that is also associated with a reduction in under-5 years mortality, and a decrease in unproductive health spendings. Thus, these underpinnings are confirming that financing model of health system is not primarily an economic issue, but one of well-organized public governance, ensuring the system’s resilience and sustainability over time. In contrast, countries with inefficient institutions, lack of transparency, and incoherent budgetary mechanisms fail to convert available resources into real improvements in health outcomes, regardless of the level of funding Gabani et al. (22).

At the same time, Hawkins et al. (24) highlight the important role of the institutional framework in the resource allocation process. The findings reveal that countries that have introduced standards for financial accountability, internal audit, and public reporting have succeeded in reducing fiscal inefficiency, and to distribute resources toward priority niched areas, respectively preventive health and medical infrastructure. Consequently, funding becomes an institutional instrument that ensures the synergy between public policies and health outcomes. Rotulo et al. (27) states that fiscal decentralization, when supported by efficient governance, can improve the provision and accessibility of health services, highlighting that the transfer of financial responsibilities to local authorities enables for more flexible allocation of funds and promptly adaptation to community needs. In contrast of substantial administrative capacities, the local decentralization can lead to imbalances and inequalities fostering once again the theoretical background according to which performing healthcare systems rely on a large extent on how public resources are managed. Nevertheless, funding mechanisms which are characterized by transparency and results-oriented, the impact on public health is considerable, reflecting in access to high-quality services, inequities being reduced, thus strengthening the long-term sustainability. Therefore, financing directly mediates the relationship between good governance and public health, ensuring alignment between institutional decisions and the needs of society.

Institutional Theory is central to our research, as the study examines the balance between good governance, health systems resilience, and long-term health financing priorities across the EU regions.

Based on the above theoretical explanation and the multidimensional framework of good governance, health status, and financing performance as forces influencing health systems outcomes, the following research hypotheses were developed, as follows:

*H*1. The relationship between good governance and health status is conditionated by the level of funding for the healthcare system.

*H*2. The quality of public governance has a significant positive/negative association with the health status of the population and health financing.

Furthermore, in order to enhance the theoretical framework, a systematic review of the literature was employed, focusing on how public governance and financing mechanisms influence the performance of health systems in the European Union. A systematic review allows for a structured approach to the scientific literature, based on clear and objective criteria. The purpose of this analysis was to identify, select, and evaluate relevant scientific studies, thereby reducing the influence of subjective factors and the risk of drawing conclusions based on incomplete or distorted information. Thus, systematic review provides a more accurate and comprehensive literature review, as it relies on rigorous procedures for selecting and excluding sources, thereby excluding irrelevant information. Unlike a narrative or traditional review, it aims to minimize errors and provide an objective synthesis of existing knowledge, as also confirmed by Lobonț et al. (30).

Through the systematic review we captured how public governance policies influence public health systems through the intermediary role of funding. The analysis also seeks to identify major trends, the theoretical approaches applied in research, and the main future directions in which the field has developed in recent years. The scientific articles were identified and selected using the Web of Science Core Collection platform. To obtain the most accurate and robust results, keywords were integrated using Boolean/Phrase operators, such as “public governance” and “public health” and “financing.”

**Figure 1:**
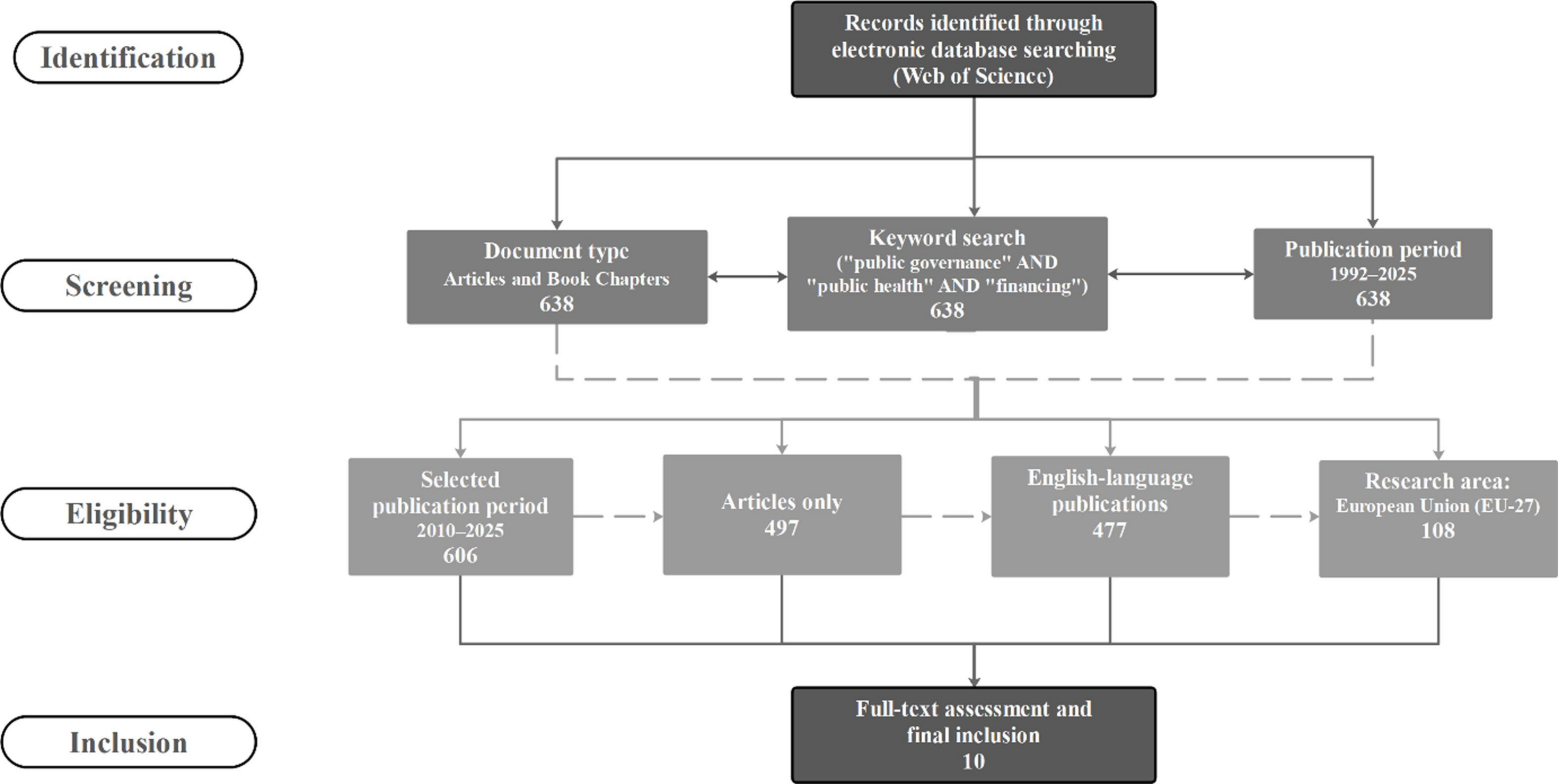
The framework of process selection—final sample SR. Source: data extracted and analyzed by the author from WoS (Web of Science) in EdrawMind.

To focus the analysis on a more relevant period and to capture recent trends in public health and governance in the European Union, the timeframe covered the period between 2010 and 2025, considering only scientific articles, and to ensure linguistic consistency and comparability of the studies, the selection included only documents published in English. Although the final research documents included were selected after a well-defined criterion, thus many articles did not meet the specific requirements of the research. Following this rigorous evaluation, the final number of articles relevant to the in-depth analysis was reduced to 10 scientific papers. The exhibit the complete selection process for the final sample of the 10 articles we schematically represented it below in Figure 1.

The overview of the final sample of research documents included in the analysis are highlighted in Supplementary Table S1.

Considering that healthcare infrastructure and its efficiency, as a result of proper budget allocation within the context of the European Union, have improved over the past two decades, with a focus on transparency and optimization at the institutional level. Recent studies analyze this phenomenon, examining the quality of governance in relation to funding and whether these factors directly or indirectly influence the health status of the population.

Santandrea et al. (31) examine public-private partnerships (PPPs) in the healthcare sector in the United Kingdom. The research aims to highlight the main weaknesses associated with these financing models and is based on a systematic review of the specialized literature published between 1990 and 2014, processed through computer-assisted content analysis. The findings reveal three major areas of concern: the financial structure of projects, the incomplete transfer of risk factors to the private sector, resulting in the state retaining ultimate responsibility, and contractual rigidity, which reduces the healthcare system’s ability to adapt to demographic, technological, and service organizational changes. In the same period, Ferrelli et al. (32) described the implementation of an initiative to strengthen governance in Italy and Egypt’s health system, aimed at enhancing the managerial skills of health professionals and modernizing decision-making processes within the health sector. The initiative, carried out between 2010 and 2015 under a cooperation agreement between Italy and Egypt, utilized an educational methodology based on PBL (problem-based learning), in which a select group of specialists from hospitals and healthcare systems was trained to, in turn, become capable of passing on these skills. They participated in an intensive applied learning program in the field of health governance and were subsequently responsible for expanding and replicating the course to other institutions and specialists involved in the organization of the health system. The project established a specialized governance unit, which helped to create a collaborative network among public and private health organizations and to introduce continuous improvement practices.

Duran et al. (33) reveal that, although the legal framework provides for recently established accountability mechanisms, these are insufficiently applied, and managers have limited autonomy in relation to the budgetary and operational tasks assigned. Vulnerabilities and low levels of funding appear to be a key factor in the extension of outstanding payments, and the difficulty hospitals face in increasing their productivity levels. A year later, Johansen et al. (34) analyze the transformations in Slovenia’s primary healthcare system, highlighting how reforms implemented after the 1990s improved the accessibility and quality of services in a system characterized by universal coverage. Evidence shows that, despite health expenditures lower than the European Union average, Slovenia has achieved superior health outcomes, reflected in a decline in infant mortality, a reduction in premature deaths, and life expectancy on par with the European average aspects that confirm the effectiveness of a prevention-oriented approach.

Waitzberg et al. (1) analyze the responses of health systems in seven Mediterranean countries (Cyprus, Greece, Israel, Malta, Italy, Portugal, and Spain) during the first 6 months of the COVID-19 pandemic, given that these countries started with relatively limited medical resources. An important common feature is that all countries have guaranteed universal access to COVID-19 services, including for groups without prior health coverage, thereby ensuring the continuity of population protection. At the same time, Ussai et al. (35) examine the major mental health challenges facing young people in Italy in the post-pandemic context. The study employs a methodology based on a systematic review of national and regional policy documents, operational programs within the Italian healthcare system, and recently published literature on the effects of the COVID-19 pandemic. The results highlights that investments in youth mental health have been limited and poorly planned, with many interventions being temporary and financially disorganized. Perez et al. (36) examine global health governance through public-private partnerships (PPPs) and the institutional links between organizations involved in the formulation and coordination of health policies. The results reveal that the concentration of decision-making in one organizational elite can lead to structural imbalances and the risk that certain health priorities will be insufficiently investigated.

Țăran et al. (37) address one of the most compelling issues in contemporary European health policy: the relationship between the quality of public governance and the performance of healthcare systems, including all European Union Member States for the period 2010–2021. The main findings emphasize the need to strengthen governance mechanisms, also discussing the importance of developing monitoring frameworks capable of assessing both institutional progress and quality outcomes for the society. Nevertheless, Ridde et al. (38) examine how the funding challenges caused by the COVID-19 pandemic have affected the operations, focusing on the impact on healthcare staff and patients’ access to services. Results reveal that medical staff experienced demotivation due to the late payment of salary incentives and increasingly difficult working conditions. In addition, patients often incurred unexpected costs for initial diagnostic services, which undermined equitable access to care.

Berardi, et al. (39) analyze the reforms implemented in the health care systems of the G7 countries. The findings reveal that responses to the financial crisis focused primarily on strict cost control through the recentralization of decision-making and restrictive budgetary measures, which in some cases placed additional pressure on the population’s access to healthcare services.

Based on the results of the systematic analysis, we observe that there are differences among countries in the performance of their health care systems, depending on the characteristics of resource allocation to the healthcare sector, which are influenced by the quality of public administration, with European countries facing challenges such as: (i) challenges in terms of funding and governance, even if reforms have been implemented, their effectiveness has been limited due to institutional imbalances (40); (ii) differences in the performance of medical infrastructure, which stem from the objectives of public policies and the performance of governance (41).

*H*3. The strength of the relationship between good governance and health status varies across EU-27 Member States; it is relatively higher in countries with higher financing level.

Moreover, given the ongoing transformations of health systems in the European Union, public governance is a key factor in strengthening the effectiveness and resilience of health policies. At the same time, financing plays a central role, acting as a mediator between governance mechanisms, their implementation, and the outcomes achieved at the population level. To capture how these concepts are addressed and interconnected in the existing comprehensive literature, a complex and in-depth bibliometric analysis was conducted to highlight major trends and significant contributions in the research field.

**Figure 2:**
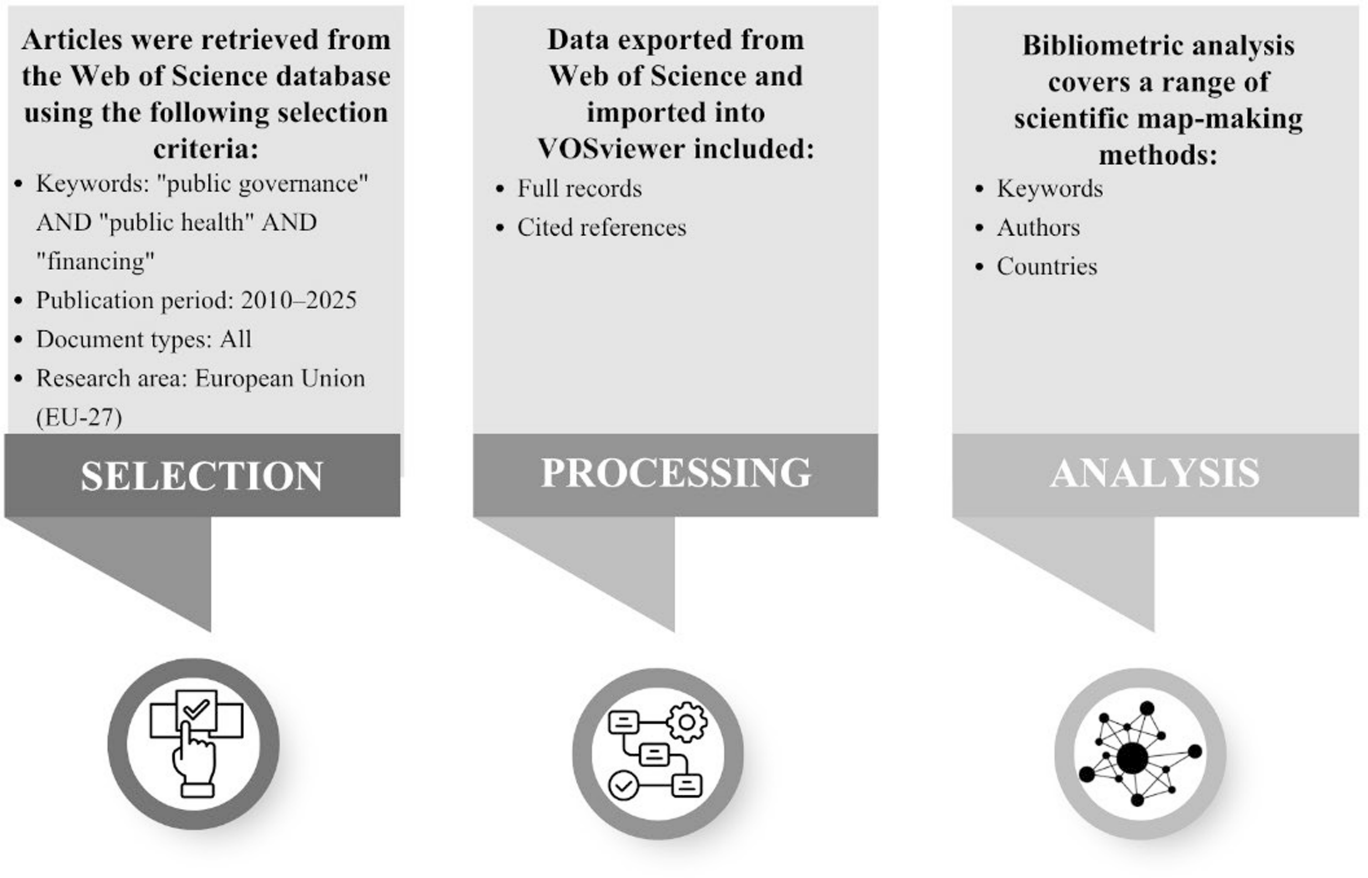
Data selection and processing for VOSviewer analysis. Source: author’s own processing in Canva 2025, Suite 2.0.

Figure 2 depicts the stages for the bibliometric analysis, illustrating the steps to identify, select, and process articles from the Web of Science database, as well as the subsequent configuration of the analysis in the VOSviewer software.

**Figure 3:**
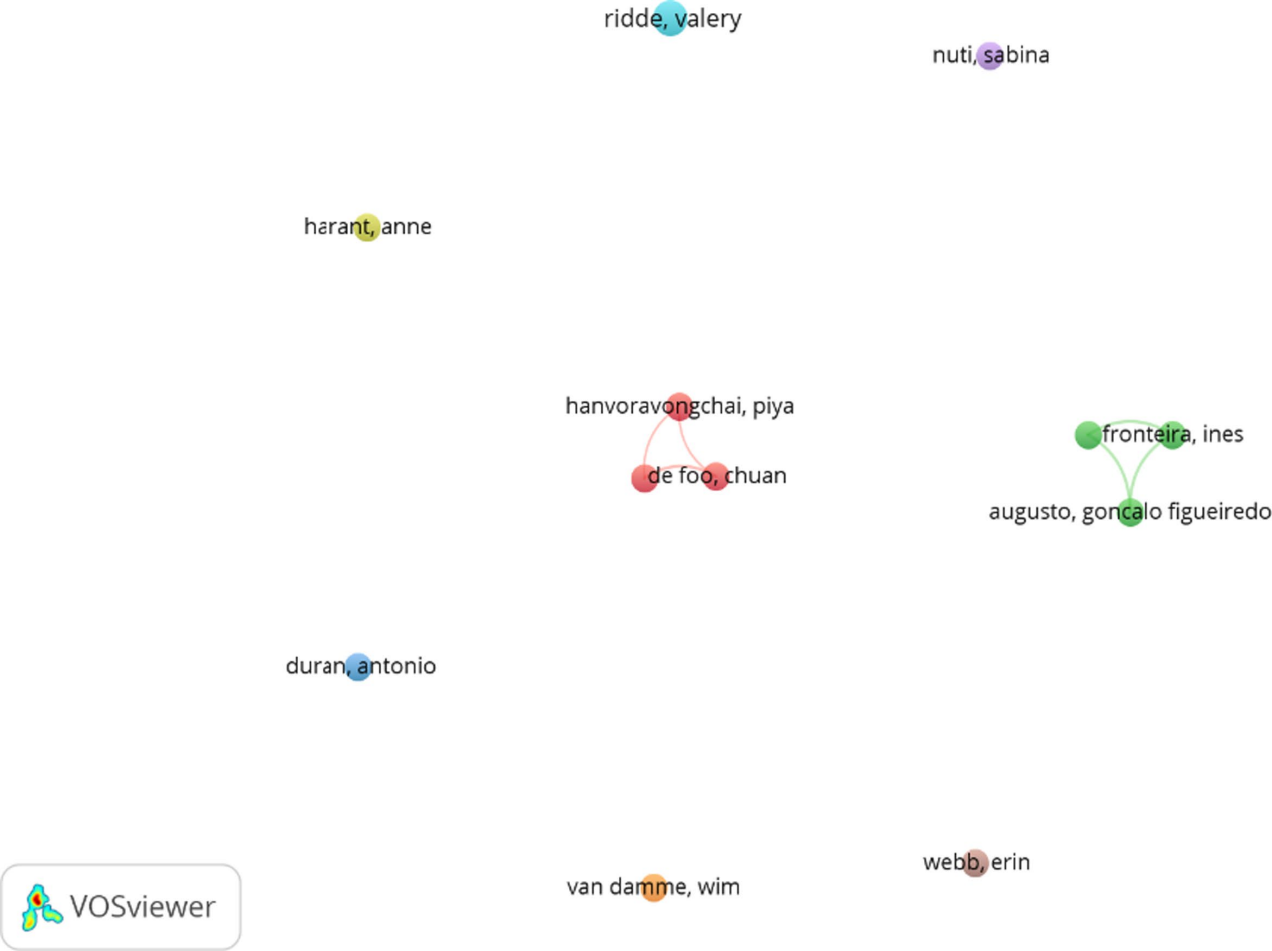
Co-author network based on the number of publications by each author. Source: data processed using VOSviewer version 1.6.20.

Keyword analysis allows the identification and interpretation of the most recurrent terms in the selected literature. The frequency of these keywords provides insight into the central themes addressed by the authors and the predominant research directions in the field. Although the search was initially built around three main terms (“public governance,” “public health,” and “financing”) the network generated by VOSviewer also highlighted other high-frequency concepts suggesting a broader thematic structure. Among these terms the most prominent words are: “COVID-19” (with 12 occurrences) “care” (9 occurrences) and “health systems” (with 8 occurrences). Figure 3 shows the most relevant keywords identified after applying a minimum threshold of 3 occurrences.

**Figure 4:**
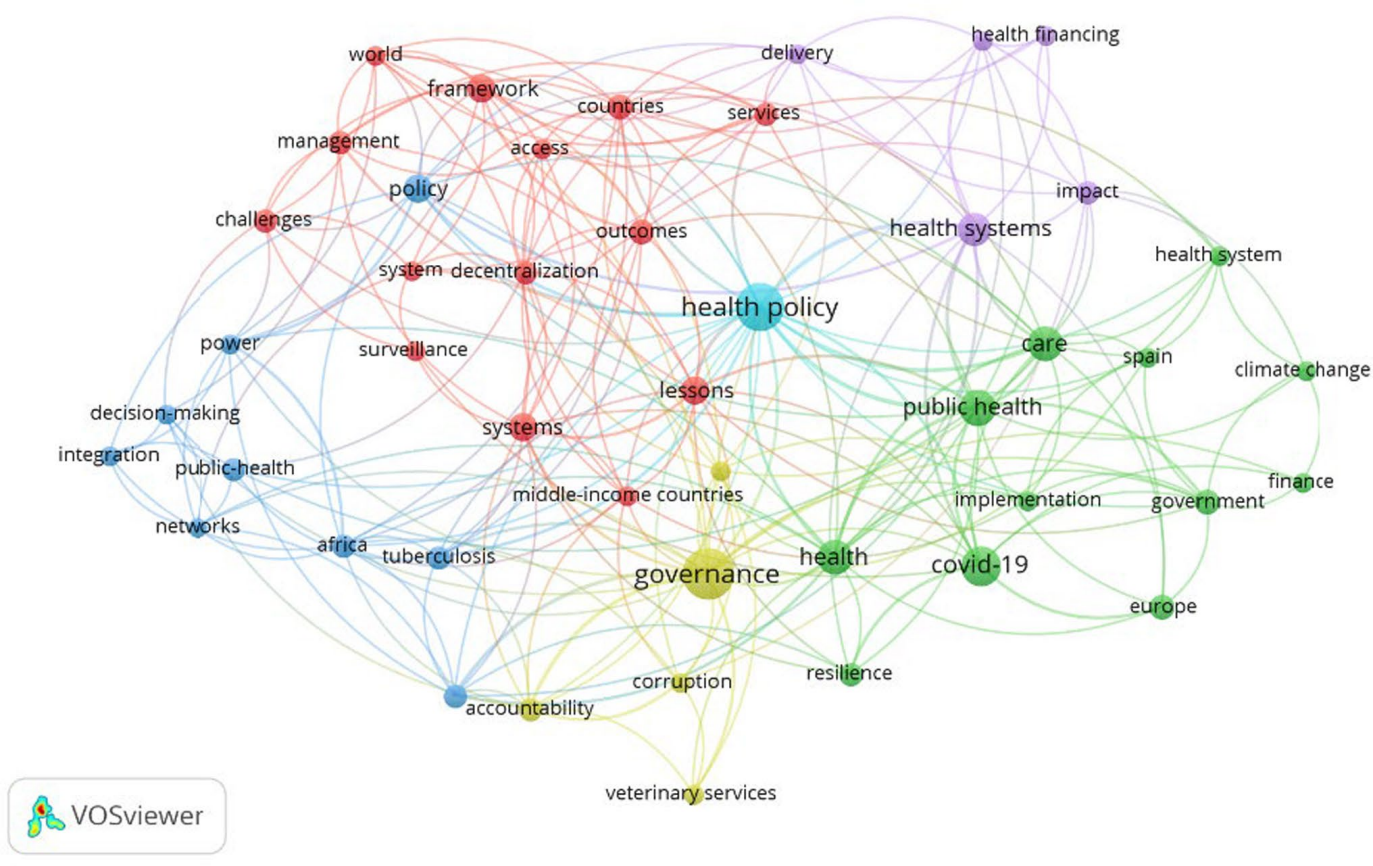
Key terms map—network analysis. Source: data processed using VOSviewer version 1.6.20.

The Figure 4 results reveal that good governance plays a pivotal role in how health systems operational process, influencing both their institutional architecture and the outcomes achieved. Supplementary Table S2 presents all six clusters, with associated key terms. The main findings highlighted strong connections between the organization and management of health systems (red group), as well as to the one focused on financing (purple), suggesting that the level of accountability, transparency, and public oversight determines the quality of financial resource usage. Within the European Union, health system financing operates as a link between the governance framework and the performance of health services, as decisions regarding budget allocation, decentralization, and the prioritization of public spending directly influence the systems’ ability to meet the health needs of the population. Ultimately, many clusters have focused on health and public health policies (green and turquoise groups) suggest that system performance depends not only on the volume of funding, but also on how it is managed within the existing institutional framework.

The co-authorship network (Figure 3) reveals a fragmented structure that is characteristic of fields with a pronounced interdisciplinary nature.

Among the results obtained in Figure 3, two groups show clear collaborations: the red cluster, consisting of De Foo C. (61 citations), Hanvoravongchai P. (80 citations), and Legido-Quigley H. (61 citations), and the green cluster, consisting of Augusto G. F. (53 citations), Fronteira I. (53 citations), and Simoes J. (53 citations). The other clusters include authors isolated within the network, such as Duran A. (44 citations), Harant A. (56 citations), Nuti S. (110 citations), Ridde V. (15 citations), Van Damme W. (33 citations), and Webb E. (106 citations). Supplementary Table S3 presents details regarding the number of documents, citations, and link strength. Nuti et al. (42) analyses the Italian health system in a context of significant decentralization and diverse regional governance models. The results reveal that evaluating regional performance within a common framework, based on standardized indicators, and ensuring transparency through data publication contribute to a meaningful improvement in the capacity to generate healthcare outcomes, provided these are integrated into regional governance mechanisms. Duran et al. (33) show that the managerial autonomy of public hospitals is limited by a dysfunctional financial framework, in which budgetary responsibilities are not supported by adequate resources. Chronic underfunding and the inadequate application of accountability mechanisms lead to the accumulation of arrears and poor institutional performance, even in the presence of formal governance reforms. Similarly, Ridde et al. (38) highlight the critical role of public funding in ensuring the resilience of health infrastructure during crises. The authors demonstrate that delays and disruptions in financial flows directly affect access to services and equitable care, particularly in contexts characterized by high reliance on out-of-pocket payments by patients. The findings show that funding is not merely a technical resource but a governance tool capable of mediating the relationship between public policy and health outcomes.

**Figure 5:**
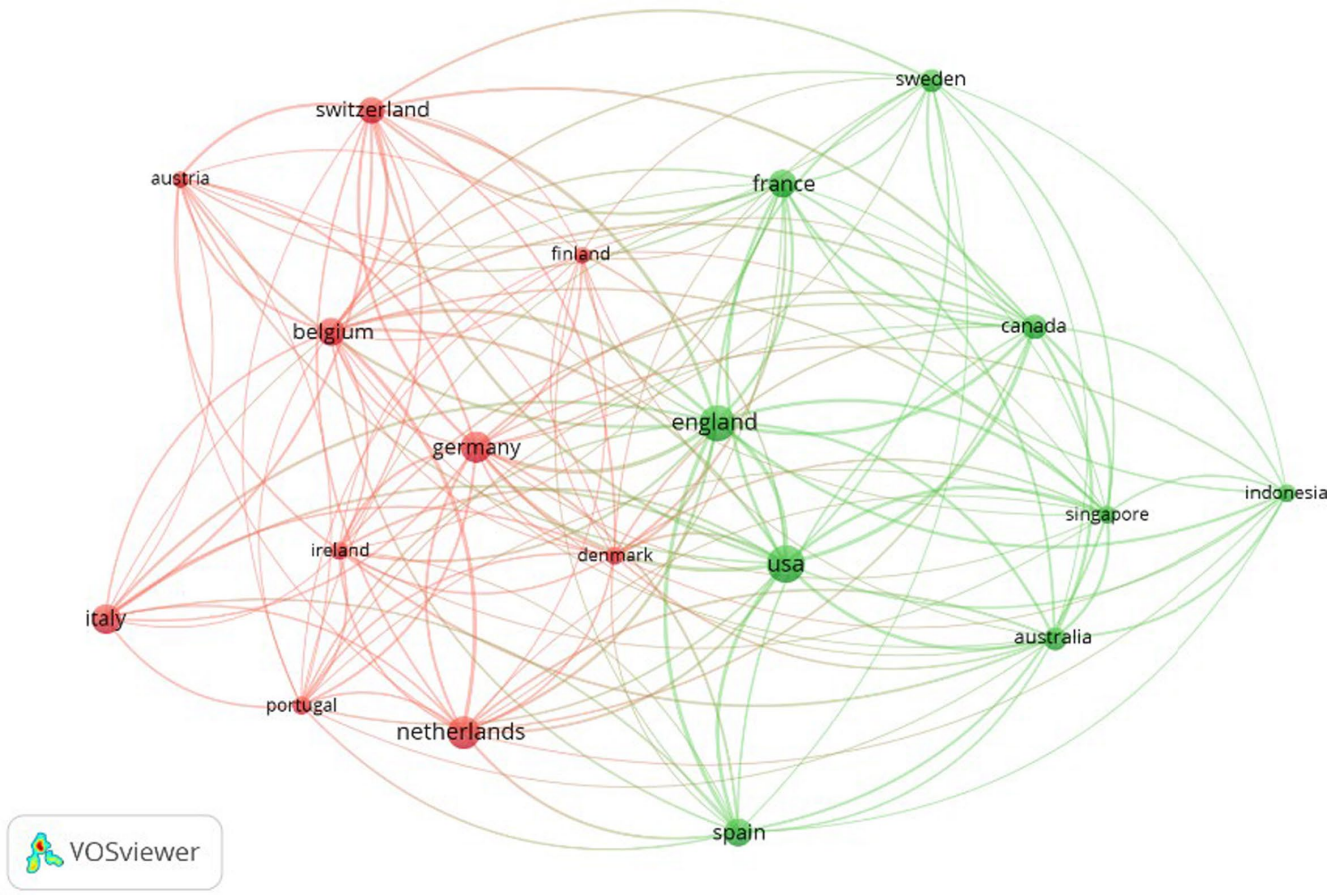
Network of scientific co-authorship among the countries involved in the publication of the papers. Source: data processed using VOSviewer version 1.6.20.

Last but not least, the country-level co-authorship analysis highlights international scientific collaborations, Figure 5 illustrating the structure of this network.

To provide a clearer framework of the country-affiliation, while Supplementary Table S4 shows the distribution of articles by country and the collaborative relationships established between them. According to the data presented in Figure 5, we noticed consistent interest in reforming and improving the governance and financing of the healthcare system. In countries such as the United States, the United Kingdom, Canada, or Australia, specialized research has supported the analysis of public policies and funding models, in the context of debates regarding the efficient use of resources and financial stability. Similarly, in European countries such as Germany, Belgium, the Netherlands, and Italy, the literature reflects recurring processes of reform in the organization of healthcare services, driven by budgetary pressures and the need to enhance the social equity of public services. Consequently, the high volume of publications and readers indicates not only academic interest but also a practical concern for the adaptation and modernisation of health systems.

## 3 Materials and methods

### 3.1 Methodology

This study aims to empirically investigate the relationships between governance quality and health outcomes, considering the mediating role of healthcare funding, within the European Union Member States (EU-27 MS). Thus, we consider identifying the influence that the quality of institutional governance has on health sector outcomes, as well as to evaluate the role of financing in this relationship. The empirical analysis of the interdependencies among the indicators was conducted using a set of advanced econometric techniques in line with the research questions:

a) Descriptive statistical analysis was performed using JASP software, which allowed for the definition of variables through measures of central tendency and dispersion. The purpose of this stage is to provide an overview of the data distribution and facilitate understanding of the structure of the considered indicators;
b) Correlation analysis—Heatmap, which illustrates a method used to assess the nature and direction of relationships between variables. In this research, correlations were calculated using Pearson’s correlation coefficient, denoted by R software, which measures the degree of linear association between two indicators.
c) Chord diagrams highlighted the relationships between the analyzed indicators, identifying the interdependencies among them. Lines illustrate the connections, and their thickness reflects the strength of the relationships. The method provides a visual representation of the interactions between governance, financing, and health status.
d) The Gaussian Graphical Model (GGM) method is an econometric technique that analyzes the conditional dependencies between the variables under study using partial correlations, based on the PCOR and EBICglasso information criteria, highlighting the strength of the relationships between them. The interdependencies among indicators are presented as networks comprising nodes representing the variables included in the analysis, which are connected by edges indicating the strength of the associated connections depending on the edge’s thickness, while their color reflects how they influence each other, respectively red means a negative relationship, and blue means a positive relationship. At the same time, these two estimation methods, namely PCOR and EBICglasso, distinguish themselves by the degree of permissiveness regarding interlinkages. The PCOR (Partial Correlation Network) method is a more flexible method whose network allows for an insightful analysis of the associations among the variables studied, in our case those related to the dimensions of governance quality, health status, and health system funding. On the other hand, the EBICglasso (Extended Bayesian Information Criterion) reduces the density of the network by filtering out the most robust connections, providing a highly accurate model that allows for conclusions based on the most significant outcomes. The undirected graph “ G=(V,E) includes a vertex set V={1,.…,p} as well as an edge set E⊂V×V ” (Williams, 2019, p. 3). Let “ $\Omega_{d}=(\omega_{\mathrm{ij},d})=\Sigma_{d}^{-1}$ for d = 1,2 be the precision matrix for $Χ={\left[x^{1},\ldots ,x^{n1}\right]}^{T}\in R^{n1\mathrm{xp}}$ and $Y={\left[y^{1},\ldots ,y^{n2}\right]}^{T}\in R^{n2\mathrm{xp}}$ . X and Y denote the data matrices (43).

Even though the Gaussian Graphical Model (GGM) is considered a robust approach for depicting relationships among various variables, in our case, relating to the dimensions of good governance, the healthcare status, and healthcare systems financing, it is essential to clarify that this econometric method involves significant limitations that must be taken into account during the process of result interpretation. The two GGM techniques described above, based on the extended Bayesian information criterion (EBIC) combined with the graphical operator (g) for minimum absolute contraction and selection (lasso) operator (EBICglasso) and partial correlation (Pcor), estimate networks based on partial correlations that reflect the relationships between variables, after controlling for the influence of the other analyzed variables (44). However, while the color and intensity of an edge in the networks indicate the nature and strength of the association between two variables, GGMs do not determine which variable is the dependent variable and which is the explanatory variable, and therefore do not demonstrate a direct causal relationship, identifying only conditional associations, without providing evidence of causality. Consequently, as noted by Epskamp et al. (45), the highlighted connections represent conditional dependencies that can be interpreted as potential conditional associations.

e) The hierarchical clustering analysis using the Ward method represents the econometric technique used to group the analyzed countries based on their performance in the areas of good governance and the healthcare sector. The results of the Ward hierarchical clustering process are represented by a dendrogram, in which countries form clusters according to their similarities, and within each cluster, countries are gradually separated based on their differences. Therefore, this method provides a robust path to track the heterogeneities among European Union countries, facilitating the identification of various economic models and public policies that lead to different levels of performance. The Ward clustering method, which minimizes the variance within clusters, has also been applied by other researchers, such as (46), showing robust results.

The final stage includes network modeling (Graphical Gaussian Model – GGMs) for conditional dependency, network structure estimation, and to model complex probabilistic relationships. We presume the GGMs instruments to better capture the architecture of causal dependency between governance, health status and financing, the direct/indirect relationships among them, alongside the network structure that derives from the partial correlation matrix, highlighting variables with a central role within the network and allowing the identification of how these indicators interact in a complex system. Additionally, Ward clustering allows grouping observations according to their similarity in the same cluster and then ranking countries according to their performance in order to further highlight specific patterns existing at the European level, being more suitable for our aim than other conventional approaches, highlighting the cross-country variations. We expect Ward clustering to better emphasize the structural similarities between the analyzed countries regarding governance performance, health status and level of financing, while we also want to identify distinct development/performance models, common profiles and models of good practice within the European framework.

### 3.2 Data

The empirical research utilized a panel dataset that encompassed the 27 Members States of the European Union. The indicators were collected from official databases, namely the World Bank—Worldwide Governance Indicators (WGI), and Eurostat, thus ensuring a high degree of data reliability and comparability. The good governance outcomes were measured using the six Worldwide Governance Indicators (WGI), and the health systems were proxied by health status and healthcare financing credentials. The analysis period was determined based on the availability of data for all indicators included in the study (Supplementary Table S5), covering the 2008–2024 time-spam, which was sufficiently extensive to capture relevant developments across European Union Member States.

The health dimension was split in two sub-dimensions in order to investigate the relationship between public governance and the performance of health systems in terms of health status and health financing, while the good governance comprise the six Worldwide Governance Indicators (WGI), as follows:

- public governance dimension, the following indicators were included: Control of Corruption (CC); Governmental Efficiency (GE); Political Stability and Absence of Violence (PSAV); Regulatory Quality (RQ); Rule of Law (RL); Voice and Accountability (VA).
- health status: Life Expectancy at Birth by Sex (LE); Infant Mortality Rate (IMR); Healthy Life Years at Birth by Sex (HLY); Self-Perceived Health by Sex, Age, and Urbanization (SPH); Hospital beds (HB); Self-Reported Unmet Need for Medical Care by Sex (UNMET).
- health system financing: Total Health Care Expenditure (THE); Current Health Expenditure per Capita (CHEpc); Current Health Expenditure (% of GDP) (CHE_GDP); Out-of-Pocket Health Expenditure (OOP).

Supplementary Table S5 offers an overview of the variables associated with the governance, health condition, and financing level.

**Figure 6:**
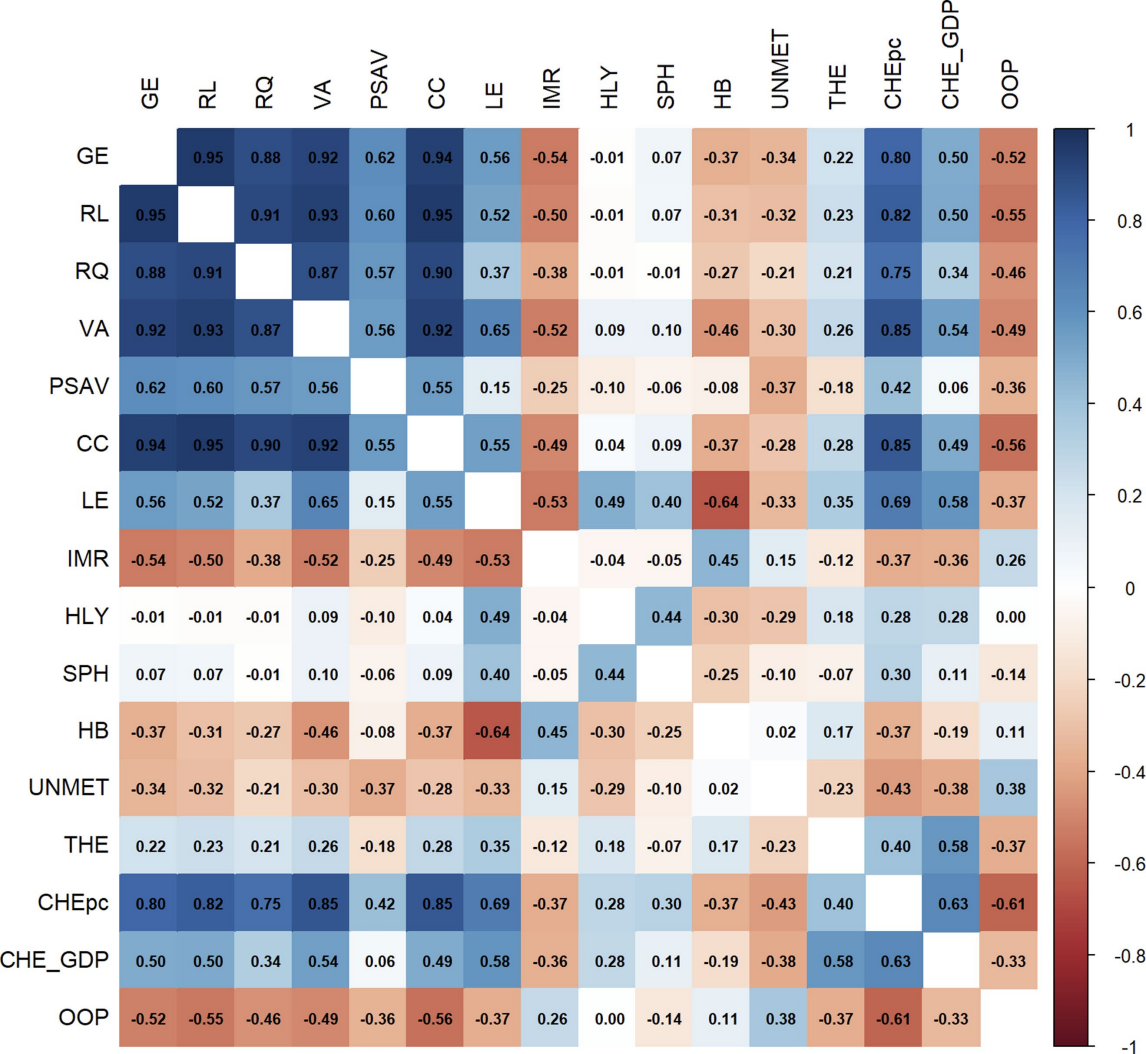
Heatmap—correlation analysis. Source: data processed using RStudio version 2026.01.2.

These descriptive statistics, along with correlation analysis and preliminary analyses, are presented in detail in Table 1 and Figure 6.

**Table 1:** Descriptive statistics for the three dimensions analyzed.

Variables	Median	Mean	Standard deviation	Skewness	Kurtosis	Minimum	Maximum
CC	0.78	0.95	0.78	0.20	−1.18	−0.38	2.44
GE	1.03	1.05	0.57	−0.18	−0.56	−0.36	2.24
PSAV	0.74	0.71	0.35	−0.42	0.17	−0.47	1.51
RQ	1.14	1.14	0.46	−0.02	−1.03	0.14	2.04
RL	1.05	1.07	0.59	−0.15	−0.99	−0.16	2.12
VA	1.07	1.08	0.35	−0.39	−0.51	0.26	1.69
LE	80.80	79.62	2.99	−0.72	−0.71	71.20	84.00
IMR	3.40	3.69	1.50	1.68	3.80	1.30	11.00
HLY	61.60	61.73	4.55	0.27	−0.15	51.40	73.60
SPH	23.05	24.60	11.20	0.60	0.14	3.90	55.10
HB	529.30	510.00	175.80	0.04	−1.21	187.40	880.60
UNMET	1.90	3.13	3.34	1.72	2.64	0.10	16.40
THE	18530.00	55470.00	94840.00	2.74	7.75	795.00	528100.00
CHEpc	2467.00	3137.00	2049.00	0.45	−1.16	401.70	8170.00
CHE_GDP	8.45	8.46	1.85	0.07	−1.04	4.48	12.72
OOP	19.42	20.89	9.13	0.78	−0.12	8.39	47.74

Source: data processed using JASP software.

Table 1 highlights the descriptive statistics of the indicators employed in our research. The data exhibits high level of variability, indicating a heterogeneous character of the European Union Member States (EU-27 MS) embedded in the analysis. The sample consisted of 459 observations, and before applying the specific methods we proceeded to the standardization of the considered data in RStudio software, were needed.

The results of the correlation analysis are presented in Figure 6, comprising the correlation map.

Figure 6 highlights strong positive correlations between governance indicators, such as Government Effectiveness (GE) and Rule of Law (RL) (0.95), alongside Rule of Law (RL) and Control of Corruption (CC) (0.95), or Government Effectiveness (GE) and Control of Corruption (CC) (0.94), suggesting that countries characterized by lower levels of corruption and a high degree of respect for the rule of law demonstrate improved performance in terms of the stability and efficiency of public administration. There are also notable strong positive correlations between governance indicators and health financing indicators, such as Current Health Expenditure *Per Capita* (CHEpc) and Government Effectiveness (GE) (0.80), Control of Corruption (CC) and Current Health Expenditure *Per Capita* (CHEpc) (0.85), and Voice and Accountability (VA) and Current Health Expenditure *Per Capita* (CHEpc), reflecting that countries with higher levels of institutional transparency and a high level of participation at the public decisions tend to have a public sector which is strongly sustained by the governments through the financing in health investments. Also, the results obtained reflect the fact that good governance promotes more efficient management of public resources and a stronger focus on health investments. At the same time, significant positive correlations are also observed between governance and health indicators, such as Rule of Law (RL) and Life Expectancy (LE) (0.52), and Government Effectiveness (GE) and Life Expectancy at Birth by Sex (LE) (0.56), reflecting the fact that institutional performance directly influences the health conditions and well-being of society.

Regarding negative correlations, the relationship between Infant Mortality Rate (IMR) and Government Effectiveness (GE) (−0.54) stands out, as well as that between Infant Mortality Rate (IMR) and Rule of Law (RL) (−0.50), which indicates that public administration dysfunction is being reflected into a high rate of deaths among children under 1 year of age. Furthermore, the Out-of-Pocket Health Expenditure (OOP) indicator shows significant negative correlations with governance indicators, such as Out-of-Pocket Health Expenditure (OOP) and Rule of Law (RL) (−0.55), Out-of-Pocket Health Expenditure (OOP) and Government Effectiveness (GE) (−0.52), indicating that a high level of health expenditure supported by citizens for health services is a characteristic of countries with a lower level of institutional development. Another important aspect is the negative correlations among health indicators, such as the relationship between the Infant Mortality Rate (IMR) and Life Expectancy at Birth by Sex (LE) (−0.53), which shows that an increase in mortality among children under 1 year of age is associated with a decrease in life expectancy, as expected.

Thus, the results (Figure 6) indicate that a higher level of governance quality is associated with a more consistent allocation of resources to the health sector, reinforcing the role of financing as a transmission mechanism between governance and the performance of health systems. At the same time, the positive relationships between governance and health indicators, such as Life Expectancy at Birth by Sex (LE) and Government Effectiveness (GE), suggest that better efficiency of the public sector contributes to more favorable health outcomes for society. On the other hand, the negative correlations observed between the Infant Mortality Rate (IMR), Out-of-Pocket Expenditure (OOP), and governance indicators (GE, RL, and CC) indicate that lower levels of institutional development are associated with poorer health system performance and greater financial pressure on the population. Consequently, the findings confirm the importance of strong governance and adequate funding in ensuring sustainable health systems, highlighting the interdependence between these factors within the context of the European Union.

All econometric estimations and data manipulation were performed using R4.3.2 and RStudio2026.01.2. The main packages and procedures applied in order to execute the analyses, including visualization of interlinkage intensity, centrality and plot tests, network analysis and conditional dependence analysis by applying the Gaussian Graphical Model (GGMs), and Ward’s method with hierarchical cluster analysis to evaluate long-run relationships, conditional dependency structures, and country-level similarities, were implemented in these advanced software’s.

## 4 Results and discussions

**Figure 7:**
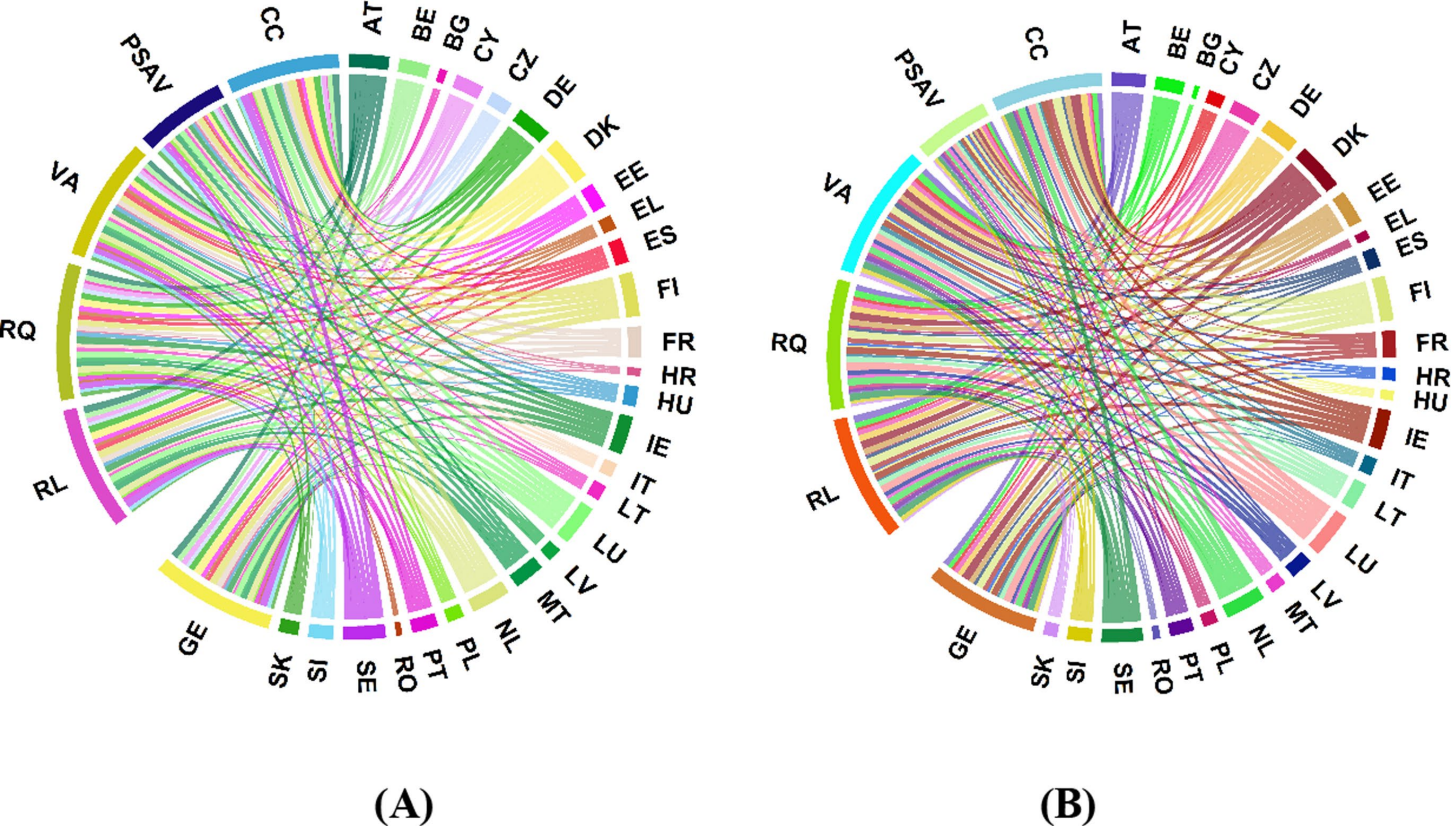
Interrelationship diagram of the public governance indicators for the EU-27 **(A)** 2008 year and **(B)** 2023 year. Source: data processed using RStudio version 2026.01.2.

To gain a deeper understanding of the interaction between governance performance factors and health condition and financing, the Chord diagram method was applied using RStudio version 2026.01.2 software to examine the interdependencies and effects among indicators. This advanced econometric method illustrates relationships between variables by arranging them in a circular format and representing the connections as arcs. Figures 7A,B illustrate the interdependencies among public governance indicators across European Union Member States (EU-27 MS), covering the years 2008 (Figure 7A) and 2023 (Figure 7B), respectively.

The first year (2008, Figure 7A) reveals strong interdependencies between indicators, and the strength of these correlations suggests that there are many divergences among European Union Member States (EU-27 MS). Countries such as Romania, Bulgaria, Hungary, Greece, and Slovakia characterized by low levels of governance quality, limited administrative capacity, and high levels of corruption have lower health outcomes, which contributes to the unfavorable condition of the population’s health. These findings are consistent with those reported by Ștefănescu et al. (47), who identified significant gaps in the management of public hospitals in Romania, including limited managerial accountability and inefficient use of resources. The authors argued that these shortcomings reduce hospitals’ ability to achieve their objectives and negatively affect the overall performance of the health care system, emphasizing the importance of strengthening institutional capacity and governance to improve the delivery of health care services. At the same time, economic constraints and limited financial resources affect these countries’ ability to develop their healthcare systems, thereby restricting access to quality healthcare services and leading to unfavorable outcomes regarding the population’s health. This result is also consistent with the findings of Mihalache et al. (48), which emphasized that healthcare financing mechanisms and the efficient allocation of resources directly influence access to medical services, the performance of the healthcare system, and, ultimately, the health outcomes of the population. In contrast, Germany, France, Sweden, and Denmark stand out for high scores on the variables considered, particularly in the Rule of Law (RL), Government Effectiveness (GE), and Control of Corruption (CC), reflecting the existence of well-structured institutions and a high level of transparency, which contribute to the development of the healthcare sector financing.

The last year (2023, Figure 7B) highlight that the intensity and complexity of connections have increased significantly, suggesting a strengthening of institutional structures and an improvement in administrative quality. Countries with lower institutional performance (Croatia, Poland, Estonia, Latvia, Lithuania) have made progress, optimizing their governance performance. Countries in Northern and Western Europe continue to show high results, reflecting a stable public administration with low levels of corruption; however, the existence of robust legal systems and the ability to implement coherent and predictable public policies also contribute to maintaining high standards.

Thus, the main results show that the analyzed dimensions over the first and last year (Figures 7A,B) reveal that differences stem from unequal levels of structural development; countries with low scores face challenges related to corruption, legislative instability, or limited administrative capacity, whereas developed countries have stable institutions with remarkable performance. Therefore, based on the results, the importance of good governance in the functioning of health systems is emphasized, and a high level of governance quality directly contributes to the health status of the citizens.

**Figure 8:**
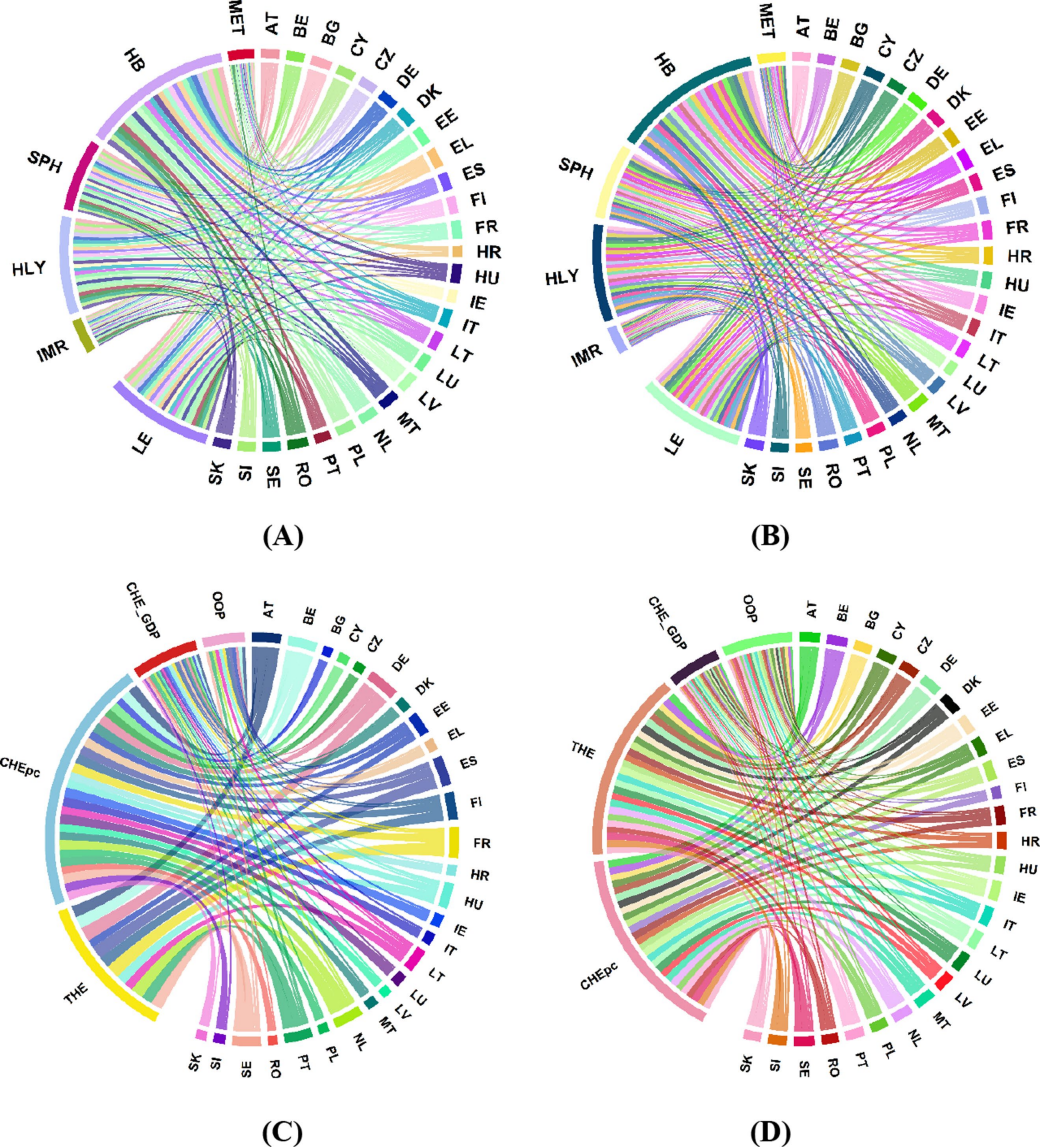
Interrelationship diagram between health status indicators: **(A)** 2008 year and **(B)** 2023 year; as well as funding indicators: The 2008 year **(C)** and 2023 year **(D)**, in the context of the European Union. Source: data processed using RStudio version 2026.01.2.

To refine the analysis, “Chord” diagrams represented health status indicators as well as financing indicators for European Union member states, analyzing the years 2008 and 2023, in Figures 8A–D.

In Figures 8A,C, countries such as Germany, France, Sweden, Denmark, Finland, Austria, Belgium, and the Netherlands stand out for their high values on both health and financing indicators. These results reflect efficient management of financial resources and a high level of health spending, directed toward the development of medical infrastructure, investments in prevention, and equitable access to health services. Consequently, these countries report favorable results for Life Expectancy (LE) and Self-Perceived Health (SPH), while the Infant Mortality Rate (IMR) is lower. These findings are supported by Ludvigsson et al. (49), who described Sweden as one of the leading countries in the EU in terms of health care spending (10.5% of GDP), alongside Germany, France, and Denmark, noting that these countries are characterized by favorable health outcomes, including high life expectancy and low preventable mortality. On the other hand, countries such as Romania, Bulgaria, Hungary, Slovakia, Latvia, Lithuania, and Croatia are characterized by low connectivity, which indicates a low level of financial resource allocation as well as shortcomings in their optimal management. Low values for health expenditure indicators (THE, CHE_GDP, CHEpc) limit the capacity of healthcare systems to provide quality services, indicating a negative association with the health indicators compared to the developed countries mentioned above. Our results are in line with those of Romaniuk and Szromek (50), who analyzed changes in health system outcomes following the implementation of health care reforms in Central and Eastern European countries and found that countries that implemented comprehensive reforms generally experienced more significant improvements in health system outcomes.

The results for 2023 (Figures 8B,D) reflect a strengthening of the connections between the analyzed variables, indicating an increase in the allocation of financial resources as well as improvements in health outcomes across the European Union. Countries with higher levels of health spending (Poland, Croatia, Estonia, Latvia, Lithuania) are associated with improvements in Life Expectancy at Birth by Sex (LE) and a decrease in the Infant Mortality Rate (IMR), suggesting that increased investment in the healthcare sector contributes to the development of its infrastructure, although effective mechanisms for resource use are also needed. In the case of Nordic and Western countries, the results are favorable, as they continue to lead with high levels of funding and the ability to translate resources into concrete health outcomes, especially after being exposed to the COVID-19 pandemic, climate change and geopolitical risks.

Based on the main findings (Figures 7, 8), distinct trends emerge among European Union Member States (EU-27 MS) regarding the performance of good governance and their relationships with healthcare systems, including also the ability to manage health resources. Developed countries benefit from well-structured healthcare systems and efficient resource allocation mechanisms, which facilitate the modernization of medical infrastructure and ensure equitable access to healthcare services for the population. In contrast, countries facing difficulties in managing funding continue to highlight the need to implement mechanisms that support health development. Based on these findings background, we can attest that H1. “The relationship between good governance and health status is conditionated by the level of funding for the healthcare system” is confirmed.

To test and validate the first hypothesis, H2, which posits an interconnection (both positive and negative) between good governance and status and financing performance of health in EU-27 countries, for the period 2008–2024, we have configured network analysis through the Gaussian Graphical Model (GGMs). The next step in this research process involves structuring the correlation networks by implementing the Gaussian Graph Model (GGM). Using this approach, a well-designed framework is obtained of the relationships between institutional quality variables, health status, and funding for this sector within the EU-27, thereby illustrating their positive or negative interlinkages. To the validation of these models, we have applied several statistical techniques as shown in Supplementary Figures S1, S2.

**Figure 9:**
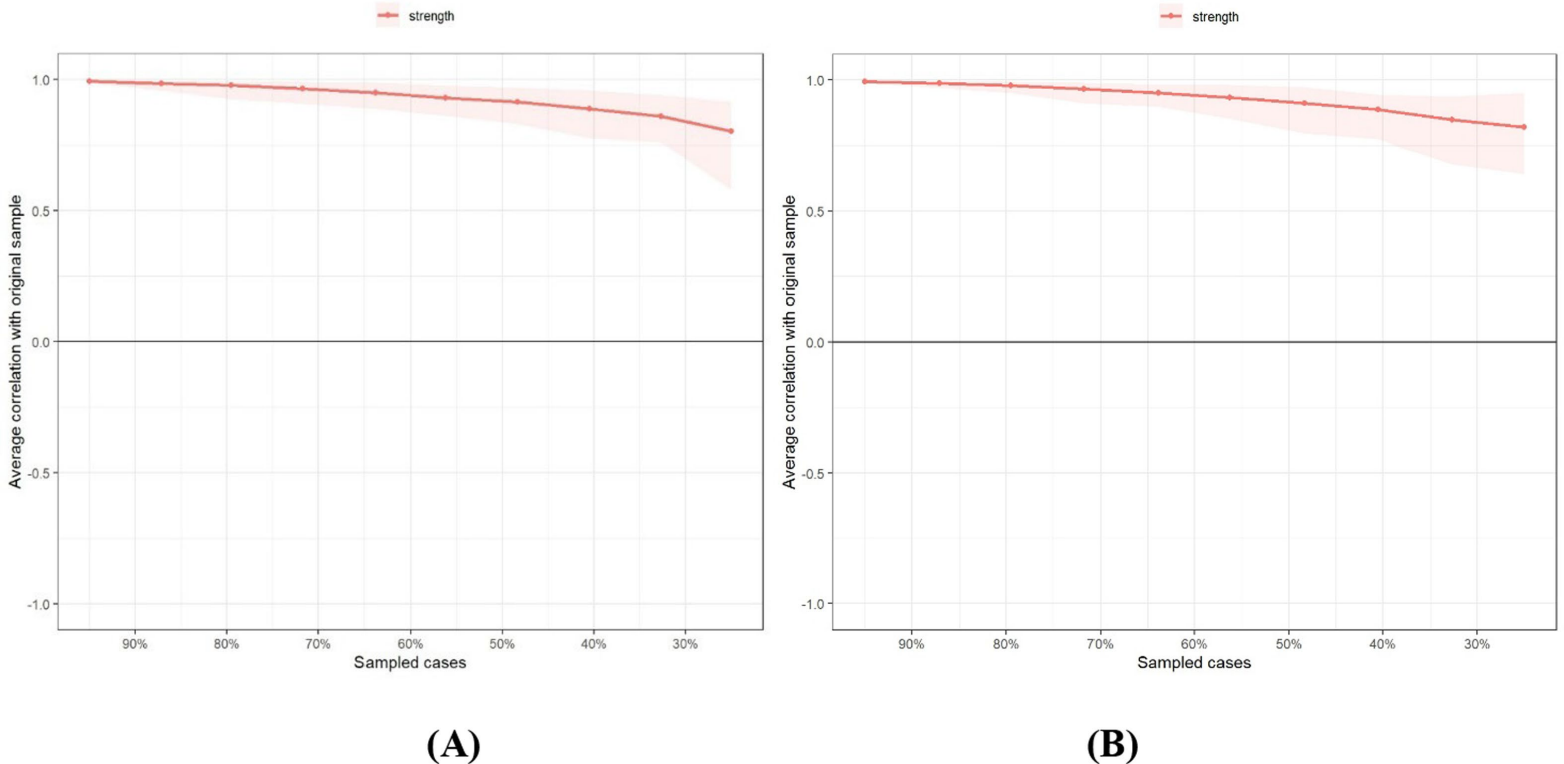
Centrality robustness—CS coefficient, Spring **(A)** and Circle **(B)** models. Source: data processed using RStudio version 2026.01.2.

Moreover, for robustness of the GGMs, we tested the Correlation Stability (CS coefficient) through bootstrap procedure. The graphical representations of the results are presented in Figure 9.

In addition, the results of the CS coeff. are presented in Table 2.

**Table 2:** Correlation stability CS coefficient, spring and circle—GGMs.

Level	Sampling levels tested					
	nPearson		Drop%		n	
	Spring	Circle	Spring	Circle	Spring	Circle
1	115	115	74.9	74.9	108	100
2	150	150	67.3	67.3	119	101
3	186	186	59.5	59.5	123	120
4	222	222	51.6	51.6	102	106
5	258	258	43.8	43.8	99	78
6	293	293	36.2	36.2	85	111
7	329	329	28.3	28.3	81	105
8	365	365	20.5	20.5	94	97
9	400	400	12.9	12.9	94	86
10	436	436	5.0	5.0	95	96

Source: data processed using RStudio version 2026.01.2. Maximum drop proportions to retain correlation of 0.7 in at least 95% of the samples: CS edge: 0.74 (Correlation Stability CS-coefficient is highest level tested); CS strength: 0.67.

The robustness of the estimated GGMs networks were assessed by applying a case-dropping bootstrap (Table 2) procedure where the stability coefficient (CS-coeff.) = 0.67 as regards the node strength and 0.74 for edge weights, both coeff. exciding the recommended threshold of 0.50 (51), confirming that our estimated network models, alongside the centrality measures are highly stable.

**Figure 10:**
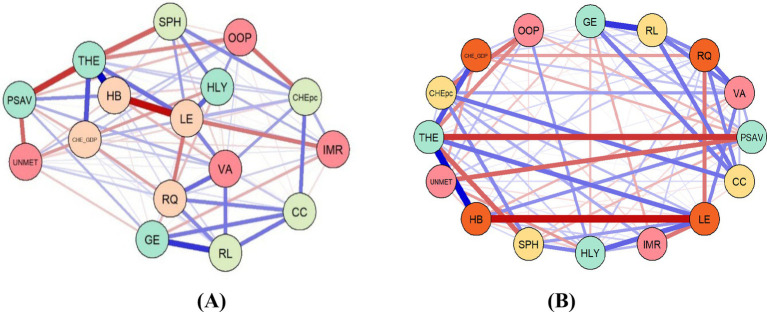
Gaussian Graphical Models (GGMs): Spring model **(A)**, Circle model **(B)**, 2008–2024 period, EU-27 MS. Source: data processed using RStudio version 2026.01.2.

Based on the nodes assigned to each indicator in the analysis, as well as the lines connecting the nodes, it is possible to visualize the interrelationships, where the intensity is represented by the thickness of the links, and their nature is reflected by colors, making it possible to distinguish between positive and negative correlations, respectively blue suggests a positive correlation, while red indicates a negative one. Furthermore, the absence of these lines signals the lack of statistically significant links. An illustration of this technique is shown in Figures 10A,B.

Applying the network model, namely Gaussian Graphical Model (GGMs), the results reveal their complex structure, with an emphasis on the direct/indirect relationships between variables. The network configuration indicates the presence of indicators with central positions, a result of multiple links/dependencies and high connection intensity.

Both models, respectively the circle-type representation (Figure 10A) and the spring-type representation (Figure 10B) capture a series of interdependencies (positive/negative) among indicators, summarized as follows:

- The findings of the GGMs reveal multiple strong links between public governance indicators, namely: Rule of Law (RL), Governmental Effectiveness (GE), and Control of Corruption (CC) and health system financing credentials, the such as Current Health Expenditure per Capita (CHE_GDP) and Current Health Expenditure per Capita (CHEpc). The analysis also observes positive synergies for the indicator number of Hospital Beds (HB) and Total Health Expenditure (THE), supporting that countries characterized by stable institutions and effective public policies have a greater capacity to financially support the medical sector, aspects that contribute to the development of health systems, yielding positive results for the population’s health status.
- The negative associations identified through GGM models highlight the link between Life Expectancy (LE) and Hospital Beds (HB), suggesting that pressures on the health system and the need to expand hospital capacity reflect the existence of persistent health problems within the population, negatively impacting its well-being. At the same time, the link between the Infant Mortality Rate (IMR) and Life Expectancy (LE) indicates poor performance of the healthcare system, associated with adverse outcomes for the general health of the population.

The relationships identified through Gaussian Graphical Models (GGMs) confirm that these models serve as a relevant methodological tool for analyzing the connections and interdependencies among indicators. Thus, the identified connections highlight the presence of significant relationships between the analyzed dimensions, stressing the importance of institutional quality in supporting and developing healthcare systems (Figure 10B). Furthermore, the findings (Figure 10A) reveal that the management of financial resources allocated to healthcare infrastructure determines its capacity, with repercussions on the health status of the population. This interpretation is supported by Mattiuzzi et al. (52), who highlighted that limitations in the capacity of the health care system (including the low number of general hospitals, physicians, and nurses) combined with high occupancy rates of acute care beds, were associated with higher COVID-19 mortality in EU countries, underscoring the importance of healthcare system capacity and organization for improving population health outcomes.

Moreover, the findings obtained through networks analysis (Figures 10A,B), respectively Gaussian Graphical Models (GGMs) identifies strong connection between Total Health Expenditure (THE) and the number of Hospital Beds (HB), as well as the associations of governance-related indicators on health outcomes, especially the existence of relevant and strong positive and negative interlinkages, which demonstrates the importance of institutional quality on the financing of health systems at the European level, as mentioned above. Our findings are consistent with those reported by Vărzaru (53), who showed that adequate funding of the health care system and strategic investments are associated with improvements in population health, including higher life expectancy and lower mortality. Furthermore, our findings are also supported by Maruthappu et al. (54), which demonstrated that reductions in public spending in the healthcare sector were significantly associated with negative health outcomes, particularly an increase in maternal mortality in European Union countries, underscoring the importance of sustained public investment in healthcare.

Following the main findings from Figures 10A,B, public funding is one of the most critical mechanisms by which governance translates into the performance of health systems. It has not only an economic dimension, but also institutional and ethical ones, as it reflects how the state assumes responsibility toward its citizens and manages collective resources. Effective public administration is characterized by intelligent management of financial resources, transparent budget allocation mechanisms, and close monitoring of results. From this perspective, funding mediates between the quality of governance and health outcomes, enabling the translation of institutional decisions into tangible benefits for the population. In addition, the positive relationships between governance indicators and health expenditure indicators that we identified using the Gaussian Graph model are consistent with those reported by Arize et al. (55), reveling that government effectiveness and public health expenditure collaborate together to improve health outcomes, highlighting the importance of an efficient public administration, combined with increased resource allocation to health care, thereby contributing to lower mortality rates and higher life expectancy. A similar perspective is also supported by Kim &Wang, who, in their study, showed that the impact of public health spending is influenced by the quality of governance, highlighting the fact that only increasing financial resources is not enough in the context of inefficient institutions that are unable to implement public policies and efficiently manage the resources allocated to medical infrastructure.

Moreover, in Figures 10A,B, we observe that the strong positive correlations between Government Effectiveness (GE) and the Rule of Law (RL), as well as between Control of Corruption (CC) and Current Health Expenditure per Capita (CHEpc), indicate that a high level of efficiency in the functioning of public institutions, reflected in higher-quality governance, demonstrates the capacity to mobilize and manage financial resources allocated to the healthcare sector and, consequently, the ability to withstand economic shocks such as the pandemic crisis (COVID-19). An important finding is the central position of the funding dimension variables within the network, from which we can infer the potential role of funding as a mediating mechanism between good governance and health sector performance. Similar findings were reported by Hadipour et al. (9), who, based on an analysis of 144 countries, suggested that both the level of public health spending and the volume of out-of-pocket payments are strongly influenced by the quality of good governance. Furthermore, the study by Gabani et al. (22) reaches similar conclusions based on a fixed-effects (FE) panel model, demonstrating the mediating role of financing by showing that economies that prioritize public funding of the healthcare system have higher life expectancy and lower infant mortality rates. Although they also point out that not every increase in funding automatically leads to better health outcomes, as its effectiveness depends on institutional architecture and the quality of governance. Thus, we can attest that H2. “The quality of public governance has a significant positive/negative association with the health status of the population and health financing” is being confirmed.

Considering H3 and for their validation, we applied a cluster analysis in order to identify groups of countries with similar patterns, pointing out those that diverge significantly from the general trends considering the year 2023, a period that allows us to observe of how the good governance is associated with the health status of citizens after the pandemic, climate and geopolitical risk, alongside the level of financing that is allocated to heath sector in the conditions after the period of macroeconomic instability. This method allows the identification of groups of countries based on the analyzed characteristics as well as the highlighting of the internal structure of the data. This analysis significantly enhances our ability to evaluate and interpret the complexity and multidimensionality of health conditions and governance performance across the European Union. As regards methodological operationalization, we used the RStudio2026.01.2 software. Based on the K-means algorithm, the data clustering procedure involves assessing the optimal number of “k” clusters and assigning each data point to its closest centroid.

**Figure 11:**
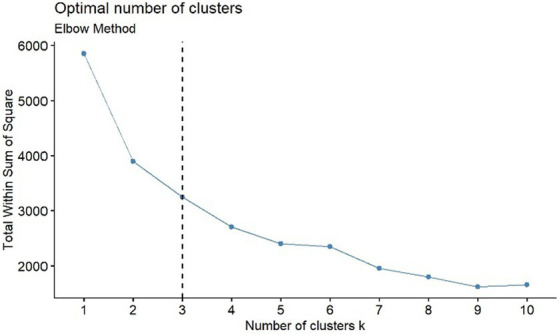
Elbow method—number of optimal clusters. Source: data processed using RStudio version 2016.01.2.

Figure 11 highlights the optimal number of clusters, and in order to determine and validate the coherence of the data cluster, we used the elbow method. Regarding the elbow method procedure, the optimal number of clusters is three, thus allowing us to observe the countries’ specific and common characteristics, alongside their behavior in facing the related context with governance performance and health conditions. Thus, our findings are supported and validated through the Elbow method procedure, which allowed us to determine the optimal number of clusters suitable for our indicators, based on the intracluster variation, thus reducing the arbitrary nature of the selection of the number of clusters.

**Figure 12:**
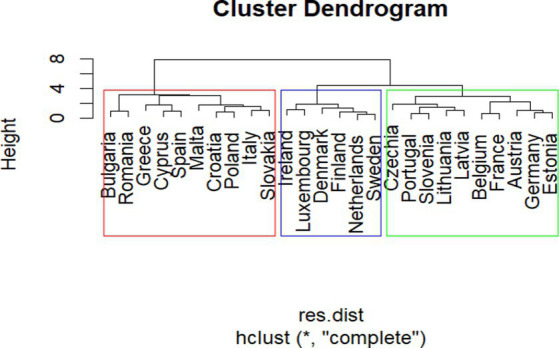
Dendrogram of the hierarchical clustering of EU-27 Member States based on public governance indicators, year 2023. Source: data processed using RStudio version 2016.01.2.

In Figure 12 we consider the Dendrogram option to document the hierarchical relationships between countries and clusters, where this method helps identify common trends among countries from the perspective of public governance indicators.

Hierarchical clustering method (Figure 12) allowed us to generate a dendrogram that identifies three distinct groups among European Union Member States (EU-27 MS). The first group (red) consists of countries such as Bulgaria, Romania, Greece, Cyprus, Spain, Malta, Croatia, Poland, Italy, and Slovakia, register a low level of institutional quality. These countries face challenges regarding the efficiency of public administration, institutional stability, and resource utilization. The second cluster (purple) brings together countries with solid administrative stability (Ireland, Luxembourg, Denmark, Finland, the Netherlands, and Sweden), distinguished by high performance in terms of the quality of public governance. The third cluster (green) includes countries such as the Czech Republic, Portugal, Slovenia, Lithuania, Latvia, Belgium, France, Austria, Germany, and Estonia, which are at an intermediate level, characterized by a relatively stable institutional framework. Our findings are broadly consistent with previous comparative studies highlighting the association between institutional quality, governance performance, and public sector efficiency across EU countries (56, 57).

Moreover, the findings in Figure 12 can be explained by a comparable level of institutional development, as well as by the ongoing process of administrative convergence. The budgetary pressures faced by these countries limit their ability to allocate resources to the health system. In addition, a weak administrative framework can create difficulties in policy implementation, directly affecting the performance of the health sector. Alongside, the hierarchy of European countries resulting from the cluster analysis (Figure 12) indicates similarities among European Union Member States regarding governance performance, due to EU-level regulations, but at the same time, there are also disparities within the sample resulting from the individual ways in which countries adapt to these regulations, this is also confirmed by researcher Farkas (58), based on the results of a cluster analysis of European countries. Thus, countries such as Germany, France, and Austria belong to the group of countries with high levels of government efficiency and stability in their regulatory and anti-corruption frameworks, facilitating the efficient management of public resources and thereby supporting long-term investments, including in the health sector. On the other hand, countries from Central and Eastern Europe, such as Croatia, Bulgaria, and Romania, continue to face deficiencies in the implementation of administrative reforms, arising from a low level of adaptability to new legislation, as well as cultural influences. Similar conclusions were reached by Majcherek et al. (59), following an analysis of the European healthcare ecosystem, highlighting how countries with consolidated institutional structures and mature governance mechanisms tend to form distinct groups from economies still undergoing reform, the differences being explained by administrative efficiency and the institutional architecture of public systems.

**Figure 13:**
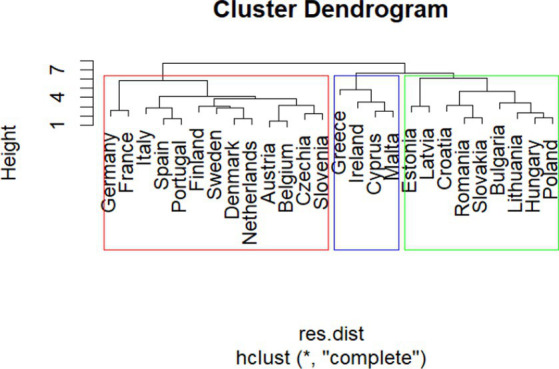
Dendrogram of EU-27 countries based on health status and health system financing, 2023 year. Source: data processed using RStudio version 2016.01.2.

Figure 13 applies clustering method to analyze the differences among European Union Member States (EU-27 MS) in terms of the performance of health system financing and its effects on health status.

As regards Figure 13, the countries in the first cluster (red) namely Germany, France, Italy, Spain, Portugal, Finland, Sweden, Denmark, the Netherlands, Austria, Belgium, the Czech Republic, and Slovenia stand out for their strong performance in health system financing, supported by institutional stability, and the ability to manage available resources to provide quality healthcare services. The countries forming the second cluster, respectively the purple one (Greece, Ireland, Cyprus, and Malta) are distinguished by specific characteristics regarding the relationships among the analyzed indicators, linked to the economic context and the structure of their healthcare systems. The third cluster (green; Estonia, Latvia, Romania, Slovakia, Bulgaria, Lithuania, Ireland, Hungary, and Poland) includes countries characterized by lower levels of financial resources allocated to the healthcare sector and structural limitations, such as low administrative capacity, which are reflected in more moderate outcomes regarding population health. In addition, the quality of healthcare services reflects the level of development of the medical infrastructure; countries with average or low levels of institutional performance face difficulties regarding access to medical services and the sustainability of their healthcare systems. Vladescu and Astarastoae (60) are also sustaining our results, considering that the low performance of the Romania’s healthcare system needs multiple reforms in health sector, revealing the need of following and adapting to the benchmark model of Netherlands which is positioned as a good performer in health status and financing of health sector by our main findings. In contrast, Nordic and Western countries are distinguished by well-managed structures, and health sector performance is higher, with a commitment to improving health systems and providing quality services to society. Thus, the data reveals discrepancies among the Member States of the European Union (EU-27 MS), highlighting the significant role that resource allocation and optimal management play in shaping healthcare systems’ resilience and sustainability. Our results are also supported by Flokou et al. (61) which also pointed out the pivotal role of health financing, positioning it as a core determinant as regards the resilience and equity of healthcare systems.

At the same time, the results (Figure 13) reveal that the largest cluster consists of countries such as France, Germany, Ireland, Sweden, Luxembourg, Finland, Denmark, the Netherlands, Austria, Belgium, Portugal, the Czech Republic, and Slovenia, being characterized by a high level of institutional quality and economic performance in the allocation of resources for the health sector. Moreover, the public policy strategy of the identified countries integrates sustainable financing, reflecting a mature model of country development focused on the process of innovation and technologization. The institutional quality of the countries positioned in the red cluster is also sustained by Doshmangir and Majdzadeh (62), which is also discussing the imperative role of good governance in enhancing the resilience of health financing. At the same time, the results obtained by Fullman et al. (63) are also attesting that countries such a Netherlands and Germany are strengthening health systems for the next generation, highlighting that the existing inequality within countries needs urgent policy attention in order to ensure that all the citizens have access to quality healthcare services. The second cluster, consisting of countries such as Bulgaria, Romania, Lithuania, Hungary, Poland, Croatia, and Slovakia, reflects an intermediate level of administrative performance, associated with a more vulnerable public health funding environment. The countries registered a lower capacity to fund the health system and, consequently, weaker efficiency in resource allocation. At the same time, the results indicate that these countries exhibit fiscal and administrative vulnerabilities, but also considerable potential for growth driven by effective good governance reforms, strengthening financing mechanisms, and ultimately facilitating these countries’ transition toward European standards. The third cluster, comprising Estonia, Latvia, Cyprus, Greece, Malta, Italy, and Spain, indicates structural similarities in institutional convergence processes and similar development models within the European Union. The budgetary pressures faced by these countries limit their capacity to allocate resources to the healthcare system. Moreover, an inadequate administrative framework may generate difficulties in policy implementations and institutional performance, thereby directly affecting the overall performance of the health sector.

The results of the cluster analysis of European countries based on health status and health financing revealed heterogeneity across the European Union, Western European and Nordic countries have emerged as top performers in this regard, by allocating significant funding for the digitalization and development of healthcare infrastructure. On the one hand, Eastern European countries continue to report poor results, partly due to moderate public investment in public health, and on the other hand, although the healthcare sector is undergoing modernization, the population lacks the necessary skills to fully utilize the available resources, thereby hindering the e-health adoption process, a conclusion also shared by the authors Luca et al. (64).

Considering the results obtained within Figures 12, 13 we can attest that the H3. “The strength of the relationship between good governance and health status varies across EU-27 Member States; it is relatively higher in countries with higher financing level” is being confirmed.

Notably, the results obtained through Chord diagrams, Graphical Gaussian Model (GGMs), and cluster analysis should be interpreted considering that structural health outcomes such as Life Expectancy at Birth (LE) and Infant Mortality Rate (IMR) evolve over longer period of time, needing a notable timespan to materialize into measurable health outcomes, thus highlighting the need to consider the lag effects in macro-health credentials, while indicators such Self-assessed Health Status (SPH), Unmet need for medical care (UNMET), and the number of Hospital Beds (HB) tend to have a relative immediate response to policy and financing changes.

## 5 Conclusion

The purpose of this analysis was to examine the relationship between the level of public governance and health systems, with funding acting as a mediating factor within the European Union (EU-27). Therefore, advanced and comprehensive methodological approach was employed to examine the relationship between quality of good governance and health conditions, respectively status and financing. The empirical research acompasses a network visualization technique of the variables, namely Chords diagrams, which facilitates the observation of variations among European Union Member States (EU-27) and the influence of the administrative and economic context on healthcare infrastructure. Alongside, the research methodology consisting of Graphical Gaussian Model (GGMs) and cluster analysis confirms that the development of health systems depends on the existence of efficient mechanisms for allocating financial resources, contributing to improvements in the population’s health status.

The Chord diagrams for the EU-27 countries allowed for a comparison of trends. The analysis highlights discrepancies between countries with well-established institutions and high administrative capacity and those facing structural deficiencies in the organization of available resources. Furthermore, the level of the most performing countries as regards the health sector can be observed through their methods of funding and supporting medical infrastructure. The results reveal that countries such as Poland, Croatia, Estonia, Latvia, and Lithuania show improvements in their indicators over the analyzed period, suggesting that these countries are making efforts to strengthen their health systems through funding. For countries that maintain a high level of development (Sweden, Denmark, Germany, the Netherlands), favorable results are evident across all years, indicating the existence of well-developed structures designed to allocate financial resources effectively. At the same time, the findings reveal countries experiencing low economic development (Bulgaria, Hungary, Romania, Slovakia) and the inefficiency of public institutions, reflecting their inability to develop the healthcare sector.

Moreover, the network visualization mapping as regards the year 2023 reveals disparities among European Union Member States (EU-27 MS) regarding the quality of public governance. Countries in Northern and Western Europe, such as Denmark, Finland, Sweden, and Germany, record high values, consistently ranking above the European Union average for all indicators, due to their well-established institutional systems. The resulting performance is directly influenced by structural and organizational factors, specifically the existence of coherent and predictable mechanisms for good governance, transparent decision-making, and effective processes regarding corruption control. All these factors have a concrete impact on the performance of health systems. Thus, the public institutions are able to allocate resources toward the development of medical infrastructure, thereby contributing to the improvement of the population’s health. In contrast, the results for Central and Eastern European countries (Romania, Bulgaria, Hungary, Poland, and Greece) show much lower scores in the area of public governance, lagging behind the European Union average, which indicates the existence of institutional limitations. Instability and limited administrative capacity, excessive bureaucracy, as well as difficulties in implementing effective governance measures, are factors that lead to public governance dysfunction, resulting in limited access to healthcare services, unequal distribution of resources, and lower quality of medical care. In this context, there are consequences on the population’s health, resulting in lower levels of well-being and quality of life.

Alongside, the results of network visualization mapping have identified disparities among the EU-27 member states regarding variations in the health status of the population. Countries such as Germany, Sweden, and France recorded values above the EU average for Life Expectancy (LE) and Healthy Life Years (HLY), as well as low levels of Infant Mortality Rate (IMR) and Self-reported unmet need for medical care by sex (UNMET), indicating a high positive association with the level of health conditions, with results influenced by a higher degree of institutional quality, which enables the optimal organization of medical services and ensures the population’s access to quality healthcare. The graph also shows that countries such as Romania and Bulgaria are below the European Union average, highlighting inefficiency in administrative capacity and the organization of healthcare systems, which limit the effectiveness of health interventions.

The Gaussian Graphical Models (GGM) results confirm the existence of both positive and negative relationships between the indicators analyzed. The central positioning of the Rule of Law (RQ), Governmental Effectiveness (GE), and Control of Corruption (CC) indicators suggest that the quality of public governance influences states’ capacity to supervise and distribute funding sources. Furthermore, the positive associations between the indicators Total Health Care Expenditure (THE), Current Health Expenditure *Per Capita* (CHE_GDP), and Hospital Beds (HB) reflect the importance of financial investments in the development of the healthcare system. In contrast, the negative correlations between Life Expectancy (LE), Infant Mortality Rate (IMR), and Hospital Beds (HB) highlight imbalances in population well-being and in the capacity of healthcare systems to effectively meet social needs. The results emphasize the importance of a competent institutional framework, as well as effective management of expenditures on health systems. However, although many research studies (55, 65) used econometric regression methods, our study employs the GGMs method, which allows us to identify conditional associations among the indicators analyzed, revealing complex patterns in their relationships. In this regard, the findings not only confirm the existing literature but also complement it, offering a broader perspective on the relationships between the quality of governance, health system financing, and the health status of the population within the EU-27.

The hierarchical clustering results indicate polarization and many discrepancies among the EU-27 Member States (EU-27 MS) regarding the performance of governance, financing, and health condition indicators, reflecting varying levels of institutional development and capacity to support the healthcare system. Among the countries of Western and Northern Europe (Germany, Finland, Sweden, Denmark, the Netherlands), the results reveal a high degree of institutional development, as well as a superior level of conversion of financial resources into quality healthcare services. A regard the good perceptions about the rule of law and the existence of functional mechanisms for managing public resources contribute to the development of medical infrastructure, as well as to improving the health of the population, reflected in part by high levels of Life Expectancy (LE). On the other hand, countries with lower performance in public governance (Romania, Bulgaria, Greece, Slovakia, and Hungary) exhibit structural vulnerabilities associated with both limitations in controlling and preventing corruption and instability in the administrative framework for implementing public policies. Furthermore, limitations in institutional coordination affect the allocation of funds to public sectors, including health systems. The consequences of these limitations are reflected in these countries’ low performance in this context, hindering access to quality healthcare.

Based on the results obtained, this study supports a series of policy recommendations. Consequently, the research highlights the need for EU-27 Member States to strengthen administrative mechanisms and optimize the allocation of financial resources within health systems, especially in the actual context of climate change and geopolitical risk. Furthermore, countries can adopt strategies focused on prevention and develop structures for monitoring and evaluating the performance of healthcare systems, with a particular attention to the sustainable use of available financial resources. These approaches can help reduce pressure on health systems and increase their resilience, thereby facilitating the population’s access to quality healthcare services. As practical policy implications, we state the need to develop continuous mechanisms of evaluation that can be used by decision-makers to ensure that the financial resources are efficiently translated into accessible and qualitative medical services.

A limitation of this study is that the employed methodological techniques are assessing, in particular, the association, interlinkages, and relationships between the dimensions considered, through the three-fold approach by employing network analysis, exploratory multivariate technique, and hierarchical clustering, thus lagging in the identification of causality effects and impact. Another critical limitation of this study is related to the potential time lag which derives from the complex processes referred to the multiple mechanisms through which improvements in governance performance and public healthcare financing reflect over certain health outcomes, such as Life Expectancy at Birth (LE) and Infant Mortality Rate (IMR). Both quality of governance and health condition and financing are dependent on the institutional context, model of national healthcare system, and policy implementation, making some health outcomes emerge gradually. In this light, the results obtained should be considered in relation to the specific analyzed timeframe, and not as reveling immediate changes in health outcomes. Nevertheless, future studies could consider dynamic modeling techniques in order to capture the temporal dynamics.

Considering the circumstances of data availability as another limitation, future studies could consider an extended timespan, including also additional explanatory variables, that would allow an in-depth analysis on sub-samples that may capture certain critical events, structural changes, and external shocks, respectively post-recession period, EU4Health Programme (2021–2027), NextGenerationEU, alongside geopolitical and energy crisis. Future research could also expand the analysis to examine the impact of investments in prevention and medical infrastructure on the sustainability of health care systems. In fact, another recommendation demands for the inclusion of indicators regarding the digitalization of medical services and the impact of modern technologies on the performance of health care systems. At the same time, another possible direction for further research involves comparative analysis among groups of countries with similar economic levels, which would allow for the identification of specific characteristics regarding the relationship between governance, financing, and health status. Furthermore, future studies could examine how countries managed the health crisis caused by the COVID-19 pandemic and the implementation of public policies regarding the allocation of financial resources to the health sector during that period.

## Data Availability

The datasets presented in this study can be found in online repositories. The names of the repository/repositories and accession number(s) can be found in the article/Supplementary material.
