# Supplementary material for: Uncovering hidden patterns: How good governance and health outcomes are shaped by the public healthcare funding?
Source: Front Public Health. 2026 Jul 30;14:1925604. doi: 10.3389/fpubh.2026.1925604 (PMC13468000; doi:10.3389/fpubh.2026.1925604)
Supplement: Supplementary file 1 [file Table_1.DOCX]

Supplementary Material

**Table S1.** Selected studies on public governance and health in the European Union, with funding as a mediating factor

| **TITLE** | **AUTHORS** | **JOURNAL** | **YEAR** |
| --- | --- | --- | --- |
| Value for money in UK healthcare public-private partnership: A fragility perspective | Santandrea, Martina et al. | Public Policy and Administration | 2016 |
| Continuous professional development and health sector governance in Egypt | Ferrelli, Rita Maria et al. | Annali dell'Istituto Superiore di Sanità | 2016 |
| Assessment of Public Hospital Governance in Romania: Lessons From 10 Case Studies | Duran, Antonio et al. | International Journal of Health Policy and Management | 2019 |
| The evolution of community-based primary health care, Slovenia | Johansen, Anne S. et al. | Bulletin of The World Health Organization | 2020 |
| Early health system responses to the COVID-19 pandemic in Mediterranean countries: A tale of successes and challenges | Waitzberg, Ruth et al. | Health Policy | 2021 |
| What is Next for Public Health after COVID-19 in Italy? Adopting a Youth-Centred Care Approach in Mental Health Services | Ussai, Silvia et al. | International Journal Of Environmental Research And Public Health | 2022 |
| Mitigating health policy fragmentation through interlocks. The networks between American and Swiss public-private partnerships | Perez, Marybel et al. | Health Policy | 2022 |
| Is The Quality of Public Governance Beneficial for The European Union Health Systems? The Mediating Role of Health Financing | Țăran, Alexandra-Mădălina et al. | Transformations in Business & Economics | 2023 |
| Financial issues in times of a COVID-19 pandemic in a tertiary hospital in Mali | Ridde, Valery et al. | International Journal of Health Planning and Management | 2023 |
| The dynamics of international health system reforms: Evidence of a new wave in response to the 2008 economic crisis and the COVID-19 pandemic | Berardi, Chiara et al. | Health policy | 2024 |

Source: authors’ proceedings

**Table S2**. Keyword groups

| **Words number** | **Group 1**  **(red)** | **Group 2 (green)** | **Group 3 (blue)** | **Group 4 (yellow)** | **Group 5 (purple)** | **Group 6 (turquoise)** |
| --- | --- | --- | --- | --- | --- | --- |
| **1** | access | care | Africa | accountability | delivery | health policy |
| **2** | challenges | climate change | decision-making | Corruption | health financing |  |
| **3** | countries | covid-19 | global health | Governance | health systems |  |
| **4** | decentralization | europe | integration | Performance | impact |  |
| **5** | framework | finance | networks | veterinary services | quality |  |
| **6** | lessons | government | policy |  |  |  |
| **7** | management | health | power |  |  |  |
| **8** | middle-income countries | health system | public-health |  |  |  |
| **9** | outcomes | implementation | tuberculosis |  |  |  |
| **10** | services | public health |  |  |  |  |
| **11** | surveillance | resilience |  |  |  |  |
| **12** | system | spain |  |  |  |  |
| **13** | systems |  |  |  |  |  |
| **14** | world |  |  |  |  |  |

Source: authors’ proceedings

**Table S3. Citation groups and papers by author**

| **Group 1 (red)** | | | **Group 2 (green)** | | | **Group 3 (dark blue)** | | | **Group 4 (yellow)** | | |
| --- | --- | --- | --- | --- | --- | --- | --- | --- | --- | --- | --- |
| Author | Doc.  /Cit. | Bond strength | Author | Doc.  /Cit. | Bond strength | Author | Doc.  /Cit. | Bond strength | Author | Doc.  /Cit. | Bond strength |
| De Foo C. | 2/61 | 3 | Augusto GF. | 2/53 | 4 | Duran A. | 2/44 | 0 | Harant A. | 2/56 | 0 |
| Hanvoravongchai P. | 2/80 | 2 | Fronteira I. | 2/53 | 4 |  |  |  |  |  |  |
| Legido-Quigley H. | 2/61 | 3 | Simoes J. | 2/53 | 4 |  |  |  |  |  |  |
| **Group 5 (purple)** | | | **Group 6 (light blue)** | | | **Group 7 (orange)** | | | **Group 8 (brown)** | | |
| Author | Doc.  /Cit. | Bond strength | Author | Doc.  /Cit. | Bond strength | Author | Doc.  /Cit. | Bond strength | Author | Doc.  /Cit. | Bond strength |
| Nuti S. | 2/110 | 0 | Ridde V. | 3/15 | 0 | Van Damme W. | 2/33 | 0 | Webb E. | 2/106 | 0 |

Source: authors’ proceedings

**Table S4.** Groups of articles by country

| **Group 1 (red)** | | | **Group 2 (green)** | | |
| --- | --- | --- | --- | --- | --- |
| Country | Doc./Cit. | Bond strength | Country | Doc./Cit. | Bond strength |
| **Austria** | 5/23 | 26 | **Australia** | 11/699 | 39 |
| **Belgium** | 15/387 | 46 | **Canada** | 12/733 | 52 |
| **Denmark** | 6/326 | 34 | **England** | 25/915 | 84 |
| **Finland** | 5/227 | 19 | **France** | 15/191 | 39 |
| **Germany** | 18/505 | 52 | **Indonesia** | 6/213 | 21 |
| **Ireland** | 6/372 | 31 | **Singapore** | 6/614 | 37 |
| **Italy** | 17/327 | 25 | **Spain** | 15/403 | 37 |
| **Netherlands** | 20/442 | 40 | **Sweden** | 10/304 | 24 |
| **Portugal** | 7/265 | 25 | **USA** | 27/969 | 73 |
| **Switzerland** | 13/137 | 46 |  |  |  |

Source: authors’ proceedings

**Table S5.** Presentation of the analysed indicators

| **No.** | **Indicator** | **Abbreviation** | **Characterization** | **Database** |
| --- | --- | --- | --- | --- |
| I. **Public Governance dimension** | | | | |
| 1. | Control of Corruption | CC | Corruption Control reflects the extent to which public power is used in the public interest rather than for private gain. The indicator highlights the effectiveness of institutions in preventing corruption and the manner in which public resources are utilized. | World Bank |
| 2. | Government Effectiveness | GE | Government Effectiveness measures the quality of public services, the capacity of the administration, and the efficiency of government policy implementation. | World Bank |
| 3. | Political Stability and Absence of Violence | PSAV | Political Stability and Absence of Violence reflect a country’s level of stability and the risk of conflicts or violence that could affect the functioning of the state. | World Bank |
| 4. | Regulatory Quality | RQ | Regulatory Quality reflects the authorities’ ability to collaborate and implement regulatory policies that support economic development. | World Bank |
| 5. | Rule of Law | RL | Rule of Law highlights the extent to which citizens trust rules, institutions, and the legal system, including contract enforcement and property protection. | World Bank |
| 6. | Voice and Accountability | VA | Voice and Accountability reflect the extent to which citizens can participate in choosing their government, as well as freedom of expression and association. | World Bank |
| II. **Health status dimension** | | | | |
| 7. | Life Expectancy at birth by sex | LE | Life Expectancy at Birth reflects the average number of years a person can expect to live, serving as a general indicator of the population’s health status. | European Commission, Eurostat |
| 8. | Infant Mortality Rate | IMR | The Infant Mortality Rate indicates the number of deaths among children under one year of age, relative to the number of live births, highlighting the quality of the healthcare system. | European Commission, Eurostat |
| 9. | Healthy Life  Years at Birth by sex | HLY | Healthy Life Years at Birth measure the number of years a person can expect to live without health limitations. | European Commission, Eurostat |
| 10. | Self-Perceived Health by Sex, Age and Degree of Urbanisation | SPH | Self-assessed health highlights individuals’ perceptions of their own health status, based on characteristics such as gender, age, and place of residence. | European Commission, Eurostat |
| 11. | Hospital Beds | HB | The number of hospital beds reflects the health system’s capacity to provide medical services and meet the population’s needs. | European Commission, Eurostat |
| 12. | Self-reported unmet need for medical care by sex | UNMET | Unmet need for medical care indicates the proportion of the population that did not receive necessary medical services, highlighting barriers to accessing care. | European Commission, Eurostat |
| III. **Health system financing dimension** | | | | |
| 13. | Total Health Care Expenditure | THE | Total Health Expenditure reflects the total amount of financial resources allocated to the health system from both public and private sources. | European Commission, Eurostat |
| 14. | Current Health Expenditure Per Capita | CHEpc | Current Health Expenditure Per Capita indicates the average amount spent on health services per person. | World Bank |
| 15. | Current Health Expenditure (% of GDP) | CHE_GDP | Current Health Expenditure as a Percentage of GDP shows the share of resources allocated to health out of a country’s total economic output. | World Bank |
| 16. | Out-of-pocket Health Expenditure | OOP | Out-of-pocket Expenditures reflect the amounts paid directly by patients for medical services, indicating the level of financial protection provided by the health system. | European Commission, Eurostat |

Source: authors’ proceedings

**Figure S1.** Centrality plot

Source: Data processed using RStudio version 2016.01.2.

**Figure S2.** Clustering plot

Source: Data processed using RStudio version 2016.01.2.
